# ATP-competitive inhibitors of PI3K enzymes demonstrate an isoform selective dual action by controlling membrane binding

**DOI:** 10.1042/BCJ20240479

**Published:** 2024-11-28

**Authors:** Grace Q. Gong, Glenn R. Masson, Woo-Jeong Lee, James M. J. Dickson, Jackie D. Kendall, Manoj K. Rathinaswamy, Christina M. Buchanan, Martin Middleditch, Brady M. Owen, Julie A. Spicer, Gordon W. Rewcastle, William A. Denny, John E. Burke, Peter R. Shepherd, Roger L. Williams, Jack U. Flanagan

**Affiliations:** 1 Department of Molecular Medicine, The University of Auckland, Auckland, New Zealand; 2 Maurice Wilkins Centre for Molecular Biodiscovery, The University of Auckland, Auckland, New Zealand; 3 MRC Laboratory of Molecular Biology, Francis Crick Avenue, Cambridge CB20QH, U.K.; 4 School of Biological Sciences, The University of Auckland, Auckland, New Zealand; 5 Auckland Cancer Society Research Centre, The University of Auckland, Auckland, New Zealand; 6 Department of Biochemistry and Microbiology, University of Victoria, Victoria, British Columbia, Canada V8W 2Y2; 7 Department of Pharmacology and Clinical Pharmacology, The University of Auckland, Auckland, New Zealand

**Keywords:** lipid kinase, membrane proteins, phosphoinositide 3-kinase, PIK3CA, protein conformation, small molecules

## Abstract

PI3Kα, consisting of the p110α isoform of the catalytic subunit of PI 3-kinase (encoded by *PIK3CA*) and the p85α regulatory subunit (encoded by *PI3KR1*) is activated by growth factor receptors. The identification of common oncogenic mutations in *PIK3CA* has driven the development of many inhibitors that bind to the ATP-binding site in the p110α subunit. Upon activation, PI3Kα undergoes conformational changes that promote its membrane interaction and catalytic activity, yet the effects of ATP-site directed inhibitors on the PI3Kα membrane interaction are unknown. Using FRET and biolayer interferometry assays, we show that a class of ATP-site directed inhibitors represented by GSK2126458 block the growth factor activated PI3Kα^WT^ membrane interaction, an activity dependent on the ligand forming specific ATP-site interactions. The membrane interaction for hot spot oncogenic mutations that bypass normal p85α regulatory mechanisms was insensitive to GSK2126458, while GSK2126458 could regulate mutations found outside of these hot spot regions. Our data show that the effect of GSK126458 on the membrane interaction requires the enzyme to revert from its growth factor activated state to a basal state. We find that an ATP substrate analogue can increase the wild type PI3Kα membrane interaction, uncovering a substrate based regulatory event that can be mimicked by different inhibitor chemotypes. Our findings, together with the discovery of small molecule allosteric activators of PI3Kα illustrate that PI3Kα membrane interactions can be modulated by factors related to ligand binding both within the ATP site and at allosteric sites.

## Introduction

Class I PI3Ks catalyse the conversion of membrane embedded phosphatidylinositol(4,5)-bisphosphate (PIP_2_) to phosphatidylinositol(3,4,5)-trisphosphate (PIP_3_), a lipid signalling product central to many cellular processes including cell growth, survival, metabolism, and migration. The class IA PI3Kα enzyme functions as a heterodimer consisting of the p110α catalytic subunit and a p85 regulatory subunit [1]. The *PIK3CA* gene encoding the p110α protein is one of the most frequently mutated genes in cancer and a driver of several overgrowth syndromes [2]. PI3K inhibitor development has provided both isoform selective and pan-isoform PI3K inhibitors [3]. While many of these block the ATP binding site, little is known about the effects these compounds have on the non-nucleotide substrate binding functions of the PI3Kα enzyme including macromolecular interactions such as the plasma membrane interaction.

**Figure 1.**
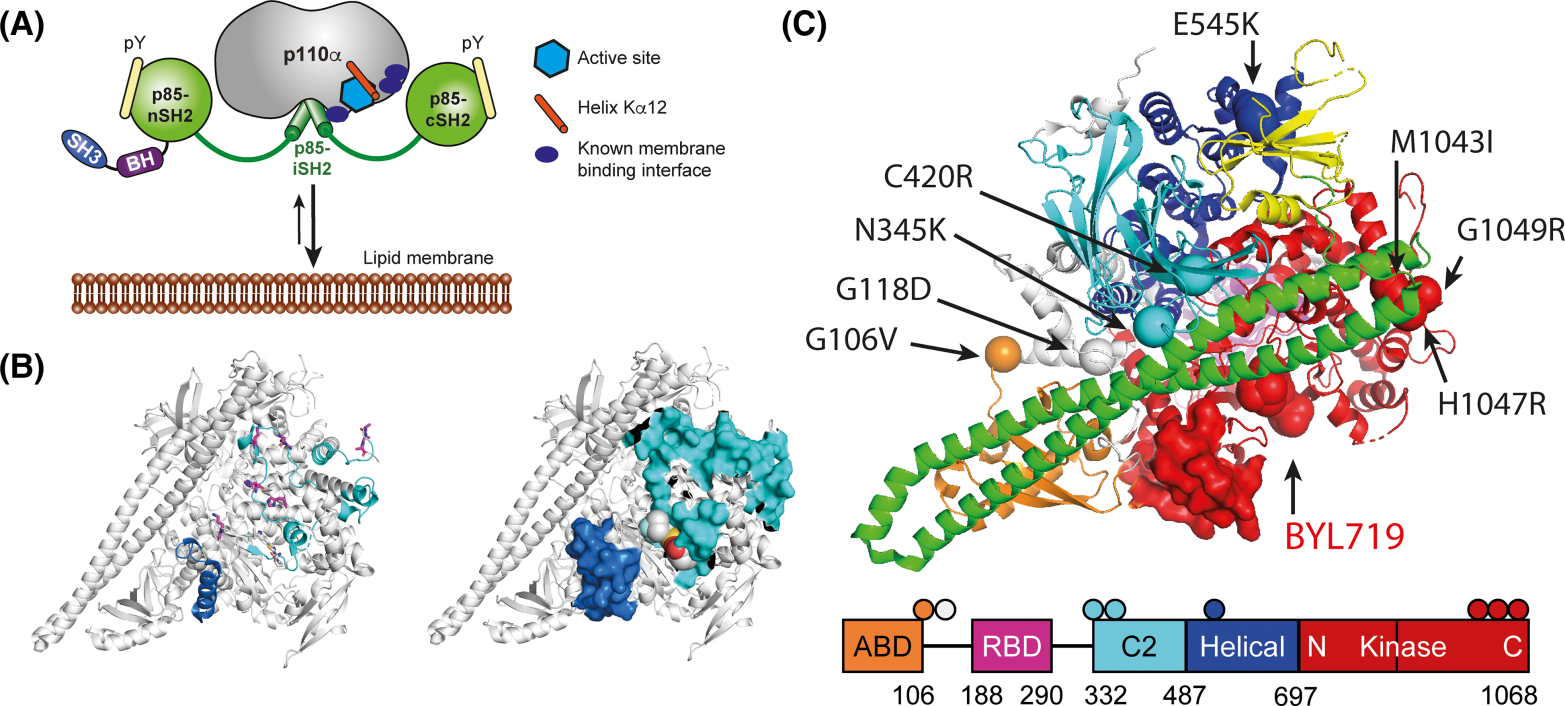
Relationship between the ATP binding site of the PI3Kα p110α subunit membrane interaction sites and oncogenic mutations. (**A**) Model of PI3Kα activation by phosphotyrosine-peptide (pY) binding to the SH2 domains of the p85 subunit, illustrating the relative positions of the active site (light blue hexagon), helix Kα12 (orange cylinder) and known membrane binding interfaces (dark blue). (**B**) Amino acids known to interact with the membrane, PIP_2_ or ATP in the PI3Kα^WT^ and PI3Kα^H1047R^ oncogenic mutant displayed on the PI3Kα^WT^ PDB structure 4jps [16]. *Left panel*: Amino acids contributing to the interaction with ATP and PIP_2_ from Ref's [5,8] are shown as magenta sticks on the 4jps structure. Regions involved in membrane binding from HDX data in Ref. [6] are coloured blue for PI3Kα^WT^ and cyan for additional regions in PI3Kα^H1047R^. *Right panel*: Surface representation of the left panel (**C**) p110α domain organisation and locations of different p110α oncogenic mutations used in this study relative to the ATP binding site occupied by BYL-719 in 4jps.

Both p110α and p85α are multi-domain proteins, and interactions between the p85α nSH2 domain and the p110α helical domain keep the PI3Kα enzyme in a low activity, basal state. SH2 domain binding to phosphorylated YXXM motifs in activated growth factor receptors and adaptor proteins [4] releases the physical constraint and allows the PI3Kα heterodimer to adopt high activity conformations with improved membrane interaction [5,6] and lipid substrate access [5,7] (Figure. 1A). Membrane binding involves a combination of electrostatic and hydrophobic interactions in the p110α kinase domain [5] with basic residues in the activation loop [5,8,9], catalytic loop [8], and hydrophobic amino acids in the C-terminal tail [5] being involved.

Cancer-associated mutations are found in both p110α and p85α proteins [10–12]. Mutations in the p110α protein mimic or enhance the different conformational states of PI3Kα^WT^ activation [6] (Figure 1B). The two most frequent p110α oncogenic mutations are found in the helical and kinase domains and activate PI3Kα through different mechanisms [13]. The p110α^H1047R^ kinase domain mutation moves the kinase domain membrane binding surface to a conformation that increases the membrane interaction of the PI3Kα^H1047R^ enzyme [14]. The helical domain p110α^E545K^ mutation mimics the growth factor activated state by blocking the inhibitory effect of the p85α nSH2 domain on PI3Kα enzyme activity and increases the interdomain flexibility needed for the membrane interaction [6]. Other p110α mutations occurring at the interface with the p85α iSH2 domain or in interdomain linkers also destabilise the rigidifying effect of p85α on p110α [6,15] (Figure 1C).

PI3Kα X-ray crystal structures show that most inhibitors occupy the ATP binding site found between the N- and C-terminal lobes of the kinase domain [5,14,16–24]. The inhibitor binding site is adjacent to protein regions that are buried at the protein-membrane interface [6] (Figure 1B), and are involved in lipid binding including the PIP_2_ substrate [8,9] (Figure 1B). PI3K structural data has also illustrated that the ATP binding site and surrounding areas can adopt different conformations in response to different ligands [5,25–27]. The potential for using this to develop compounds to specifically modulate PI3Kα activity has been demonstrated recently by the identification of a small molecule that increases PI3Kα activity and the membrane interaction by binding to a non-catalytic site [27].

ATP competitive inhibitors can regulate non-catalytic activities in some protein kinases. The effects include modulating intermolecular interactions that affect phosphatase interactions, as is the case for some MAPK family proteins [28,29], Akt [30], PKC [31] and PKD [32], or kinase dimerisation in the case of Raf [33]. ATP site inhibitors can also affect intramolecular interactions as seen by decoupling of regulatory and kinase domains in proteins with Src-family domain architecture [29,34].

As the PI3Kα kinase domain has important functional intermolecular interactions including membrane binding and PIP_2_ substrate recognition, we hypothesised that inhibitors that bind in the ATP site could also induce conformational changes that affect non-catalytic functions exemplified by PI3Kα membrane interactions. Our data show that ATP-binding site directed ligands including ATP do regulate membrane binding, and that this effect can be either negative or positive, depending on inhibitor chemistry. Benzene-sulfonamide containing agents represented by GSK2126458 have the strongest effect, reducing the PI3Kα membrane interaction and it does this by converting PI3Kα into a form that has similar properties to the basal state. GSK2126458 represents a new class of dual action inhibitors.

## Results

### ATP-site directed inhibitors regulate the PI3Kα^WT^ membrane interaction

**Figure 2.**
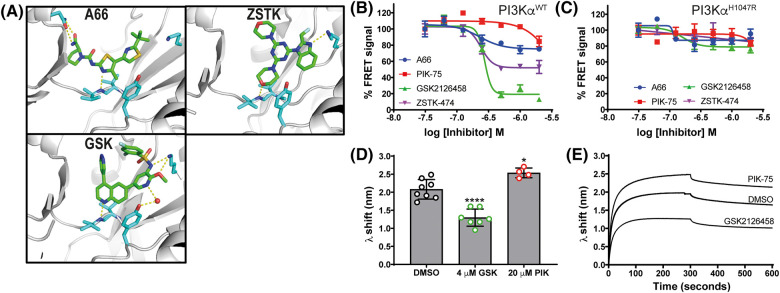
ATP-competitive inhibitors affect PI3Kα^WT^ membrane binding in FRET and BLI assays. (**A**) Inhibitors in the PI3K ATP binding sites. A66 was modelled into the active site of apo PI3Kα by molecular docking [37], ZSTK474 was crystallised in the p110δ active site (PDB entry 2wxl [25]) and GSK2126458 was crystallised in the p110γ active site with a structural water molecule shown (red sphere; PDB entry 3l08 [38]). (**B and C**) Effect of ATP-competitive inhibitors on PI3Kα^WT^ and PI3Kα^H1047R^ membrane interactions. FRET *I*-*I*_0_ values are expressed as a percentage of the FRET signal for each inhibitor at its lowest concentration. Inhibitors were tested from 2 μM down to 31.25 nM using 2-fold dilution steps. The experiment was repeated at least 2 times and data from a representative experiment is shown as the Mean ± SEM (*n* = 3). Curves were fit in Prism using a dose-response model with four parameters, including a variable Hill slope. (**B**) PI3Kα^WT^ was used at 0.5 μM with the P1pY peptide. (**C**) PI3Kα^H1047R^ was used at 0.2 μM without peptide. (**D and E**) Effect of ATP-competitive inhibitors on the BLI wavelength shift (λ) generated by 1 µM PI3Kα^WT^ binding to immobilised liposomes. (**D**) PI3Kα^WT^ liposome binding after 300 s with 5% DMSO only, 4 μM GSK2126458 (GSK) or 20 μM PIK-75 (no. of biosensors ≥4). PI3Kα^WT^ was activated with P2pY for the BLI experiments. (**E**) Representative sensogram showing association of PI3Kα^WT^ with liposome loaded biosensors over 300 s combined with a 300 s dissociation sensogram.

We selected a set of potent PI3K ATP-site directed inhibitors based on their differences in isoform selectivity and ATP binding site interactions, these included A66, PIK-75, ZSTK474 and GSK2126458 (Figure 2A, Supplemental Table S1) [25,35–39]. We examined their effects on the PI3Kα-membrane interaction using a FRET-based membrane binding assay with membrane vesicles broadly mimicking the plasma membrane composition (5% PIP_2_, 10% Dansyl PS, 20% PS, 35% PE, 15% PC, 10% cholesterol, 5% sphingomyelin) [6]. The FRET-based assay directly monitors the interaction by measuring energy transfer from tryptophan residues in PI3Kα to dansyl-labelled phosphatidylserine (PS) lipids embedded within the synthetic membrane.

We first considered if the ATP-site directed compounds could regulate PI3Kα^WT^ membrane binding in a growth factor activated state mimicked by inclusion of a bis-phospshorylated PDGFR derived peptide (P1pY) in the assay. All four inhibitors antagonised the P1pY-activated PI3Kα^WT^ FRET signal in a concentration dependent manner (Figure 2B), indicating a decrease in the membrane interaction. GSK2126458 had the largest effect with a maximum reduction in FRET signal of 85% followed by ZSTK474 and A66 with 50% and 25% respectively. The FRET IC_50_ values showed that GSK2126458, ZSTK474 and A66 were more potent than PIK-75 (Supplemental Table S2). Comparison of IC_50_ values from the FRET and enzyme inhibition data indicated that the membrane interaction effect was unrelated to enzyme inhibition. Using the basal-state PI3Kα^H1047R^ protein, we showed that the efficacy of GSK2126458 in the FRET assay was dependent on the type of PI3Kα present (Figure 2C) and not a non-specific effect of the drug.

Biolayer interferometry (BLI) was used as an orthogonal method to confirm that ATP-site directed inhibitors can regulate the PI3Kα^WT^ membrane interaction. BLI is a label-free dip-and-read method that uses wavelength changes in reflected incidental white light to report on changes in thickness of the lipid vesicles immobilised on a biosensor from protein binding. The BLI data showed that GSK2126458 could regulate the P2pY-activated PI3Kα^WT^ membrane interaction, indicated by the decrease in wavelength shift compared with the DMSO control (38%, *P *< 0.0001, Figure 2D,E). PIK-75 did not decrease the BLI wavelength shift for PI3Kα^WT^ even when used at a 1:20 ratio (Figure 2D,E), instead an increase was observed.

### GSK2126458 reduces the amount of PI3Kα^WT^ interacting with the membrane

**Figure 3.**
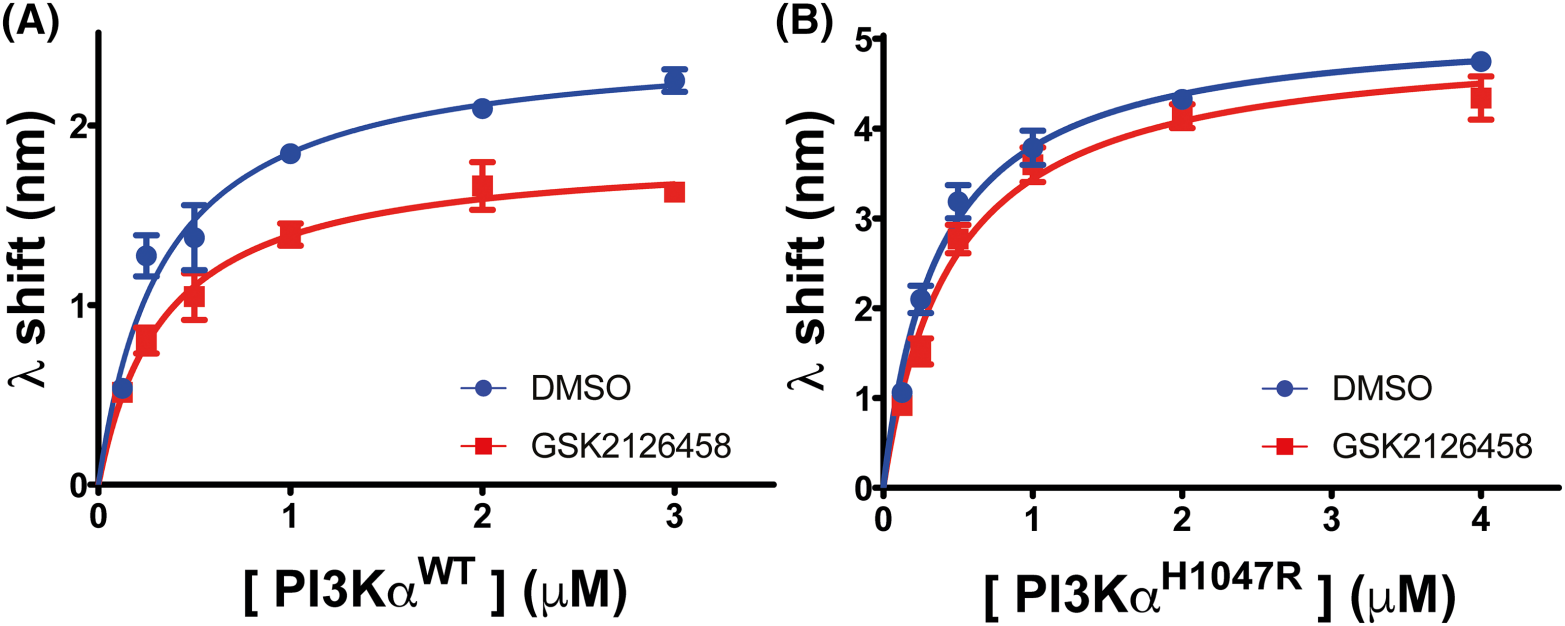
PI3Kα^WT^ membrane binding is more sensitive to GSK2126458 than the PI3Kα^H1047R^ oncogenic mutant. BLI concentration response curves for P2pY activated PI3Kα^WT^ (**A**) and PI3Kα^H1047R^ (**B**) binding to liposomes in the presence of DMSO only or GSK2126458. An inhibitor to protein concentration ratio was maintained at 4:1. Each data point is shown as Mean ± SEM (no. of biosensors ≥3). The data were modelled in Prism using a one-site, specific binding model.

To determine how GSK2126458 affected PI3Kα^WT^ membrane interaction, we obtained interaction affinity and capacity parameters for P2pY-activated PI3Kα^WT^ protein alone and in combination with GSK2126458. The data showed that GSK2126458 reduced the PI3Kα^WT^ membrane interaction capacity (*B*_max_) but did not affect affinity (Figure 3A, Supplemental Figure S1A,B, Table 1), an outcome consistent with a reduction in the amount of PI3Kα^WT^ capable of interacting with the membrane. GSK2126458 had little effect on P2pY-activated PI3Kα^H1047R^ membrane binding capacity and affinity (Figure 3B, Supplemental Figure S1C,D, Table 1).

**Table 1.** Summary of affinity (*K_d_*) and binding capacity (*B*_max_) for pY activated PI3Kα^WT^ and PI3Kα^H1047R^ in the presence and absence of GSK2126458 using BLI with mixed lipid liposomes.

	*K_d_* (µM)			*B*_max_ (λ nm)		
	**PI3Kα^WT^**					
Liposome anionic lipid composition	DMSO	GSK2126458	PIK-75	DMSO	GSK2126458	PIK-75
5% PIP_2_ + 20% PS	0.3 ± 0.1	0.3 ± 0.1	0.4 ± 0.1	2.5 ± 0.1	1.9 ± 0.1	3.1 ± 0.2
0% PIP_2_ + 20% PS	0.6 ± 0.1	0.6 ± 0.1	1.0 ± 0.1	2.2 ± 0.1	1.5 ± 0.1	3.0 ± 0.2
	**PI3Kα^H1047R^**					
Liposome anionic lipid composition	DMSO	GSK2126458	PIK-75	DMSO	GSK2126458	PIK-75
5% PIP_2_ + 20% PS	0.4 ± 0.1	0.5 ± 0.1	0.4 ± 0.1	5.2 ± 0.2	5.0 ± 0.2	5.2 ± 0.3
0% PIP_2_ + 20% PS	0.5 ± 0.1	0.6 ± 0.1	0.4 ± 0.1	5.0 ± 0.3	4.3 ± 0.3	4.4 ± 0.2
5% PIP_2_ + 0% PS	0.5 ± 0.1	0.6 ± 0.1	0.5 ± 0.1	4.7 ± 0.3	4.6 ± 0.3	5.6 ± 0.3

Data are presented as Mean ± SEM for *n* ≥ 3 biosensors. Data was fitted with a one site binding model using Prism.

### Anionic lipids affect the membrane interaction of inhibitor-bound PI3Kα enzymes

As the PI3Kα membrane interaction uses both electrostatic and hydrophobic interactions, we considered if inhibitor regulation was influenced by different anionic lipid species, including site specific interactions associated with PIP_2_.

**Figure 4.**
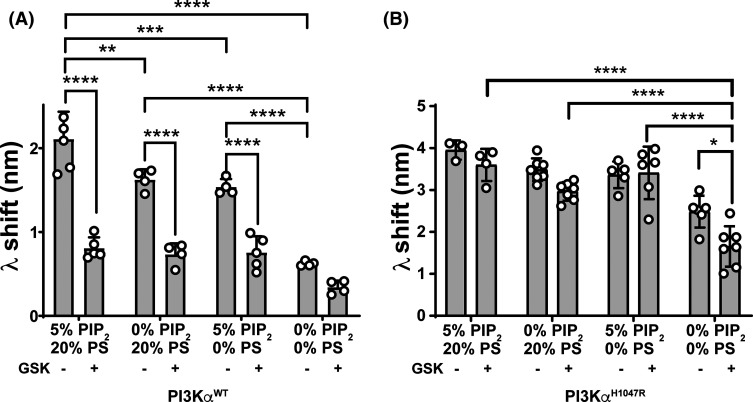
PI3Kα^WT^ membrane binding is more sensitive to GSK2126458 than the PI3Kα^H1047R^ oncogenic mutant, and the influence of GSK2126458 is unaffected by anionic lipid composition. BLI wavelength shift (λ) data for 1 µM of P2pY activated (**A**) PI3Kα^WT^ and (**B**) PI3Kα^H1047R^ binding to mixed lipid liposomes with different amounts of PIP_2_ and phosphatidylserine (PS) in the presence and absence of 4 µM GSK2126458; data is shown as Mean ± SEM (no. of biosensors ≥3).

Our BLI data agreed with studies by Hon et al. [5], and showed that apo PI3Kα^WT^ membrane binding had a large electrostatic component, indicated by a 70% (*P* < 0.0001) decrease in the membrane interaction when both PIP_2_ and PS were removed (Figure 4A). The individual electrostatic components of PIP_2_ and PS increased apo PI3Kα^WT^ membrane binding to similar levels compared with only the hydrophobic interactions. PIP_2_ also affected apo PI3Kα^WT^ affinity and interaction capacity (Table 1, Supplemental Figure S2A).

The BLI wavelength shifts showed that both GSK2126458 and PIK-75 regulated the PI3Kα^WT^ membrane interaction, but only the PIK-75 bound protein was affected by anionic lipid composition. GSK2126458 reduced the PI3Kα^WT^ membrane interaction independent of the anionic lipid composition (Figure 4A), decreasing capacity in the absence of PIP_2_ without an effect on affinity, consistent with its effect in the context of lipid membrane with both PIP_2_ and PS (Table 1, Supplemental Figure S2A). PIK-75 had a positive effect on the PI3Kα^WT^ membrane interaction, increasing capacity independently of PIP_2_ composition (Table 1, Supplemental Figure S2B,C). In contrast, the effect of PIK-75 on affinity showed some dependency on membrane composition and was decreased in the absence of PIP_2_ (Table 1, Supplemental Figure S2C).

The BLI data demonstrated that the oncogenic PI3Kα^H1047R^ mutant membrane interaction could be regulated by ATP directed inhibitors, but that this was dependent on the anionic lipid composition (Figure 4B, Supplemental Table S1, Supplemental Figure S2D–H). We confirmed the observation by Hon et al. [5] that the apo PI3Kα^H1047R^ membrane interaction had a large hydrophobic component compared with PI3Kα^WT^ as shown by only a 37% (*P* = 0.0002) decrease in wavelength shift when both PIP_2_ and PS were absent from the membrane (Figure 4B). When both PIP_2_ and PS were removed, there was also a significant decrease in wavelength shift for PI3Kα^H1047R^ in the presence of GSK2126458 (Figure 4B), suggesting that GSK2126458 could potentially affect the interaction of PI3Kα^H1047R^ with hydrophobic lipids. Affinity and *B*_max_ parameters from saturation binding experiments showed that removing only PIP_2_ unmasked the ability of both GSK2126458 and PIK-75 to negatively regulate the PI3Kα^H1047R^ membrane interaction (Table 1, Supplemental Figure S2D–G). Both compounds decreased the membrane interaction capacity, while only PIK-75 appeared to have a regulatory effect when PS was removed, and increased PI3Kα^H1047R^ interaction capacity (Table 1, Supplemental Figure S2E,H).

### Interactions with the ATP binding site are necessary for regulation of the PI3Kα^WT^ membrane interaction by GSK2126458

**Figure 5.**
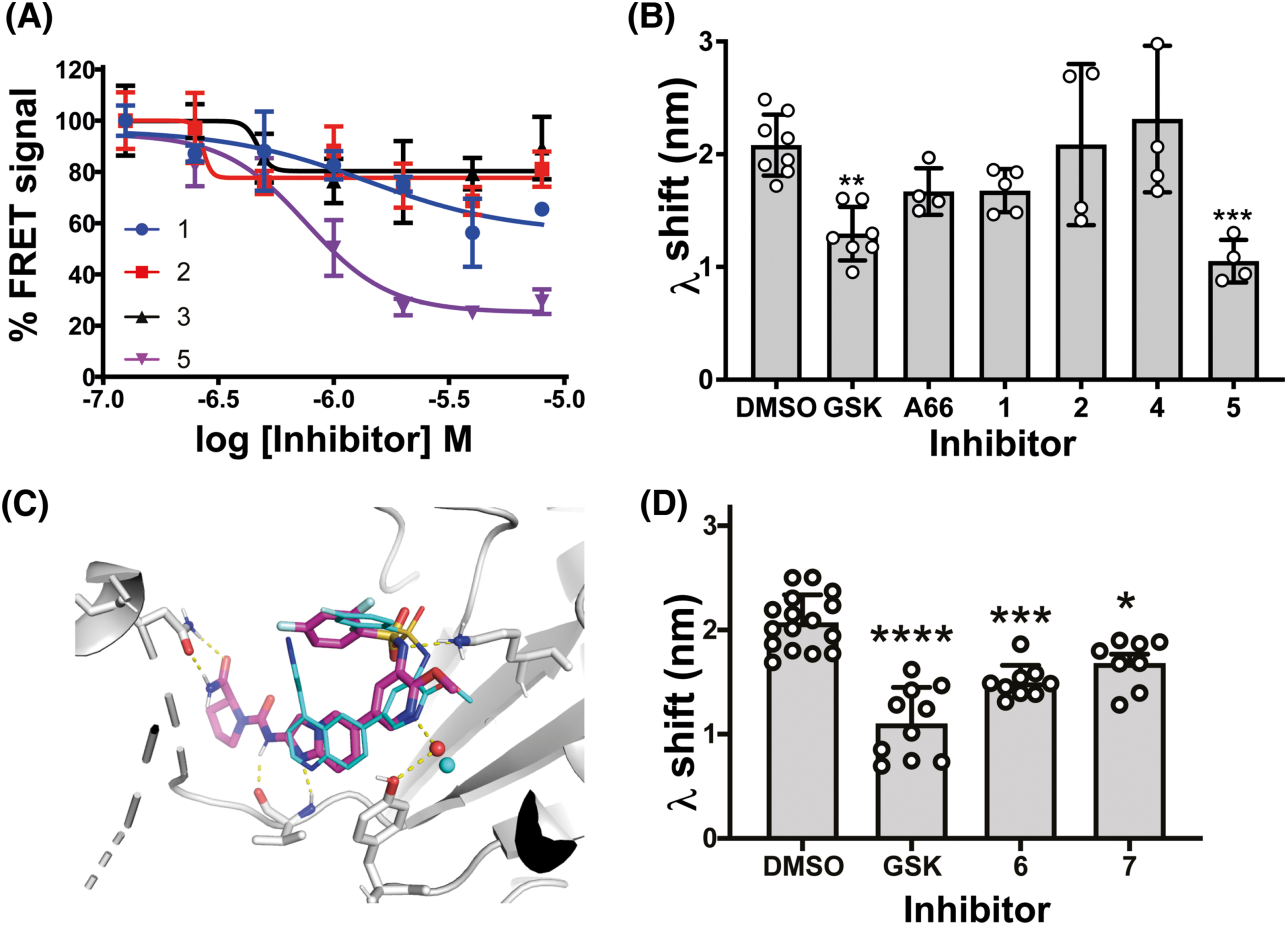
Benzene sulphonamide containing ATP competitive inhibitors are more effective at disrupting PI3Kα^WT^ membrane binding. (**A**) FRET data for 0.5 μM P2pY activated PI3Kα^WT^ in the presence of compounds **1** (blue), **2** (red), **3** (black) and **5** (magenta) across a concentration range from 125 nM to 8 μM. FRET I-I_0_ values are expressed as a percentage of the FRET signal for each inhibitor at its lowest concentration. The experiment was repeated at least twice and representative data are shown as the Mean ± SEM (*n* = 3). Curves were fit with Prism using a four-parameter dose response model with a variable Hill slope. (**B**) BLI wavelength shift (λ) data for P2pY activated PI3Kα^WT^ (1 µM) binding to immobilised liposomes in the presence or absence of inhibitor (GSK2126458, 4 µM; A66, 20 µM; compound **1**, 50 µM; compound **2**, 50 µM; compound **4**, 50 µM; compound **5**, 50 µM; *n* = ≥4 biosensors; data shown as Mean ± SD). (**C**) Molecular docking model of **5** [37] in PI3Kα^WT^ (PDB code 2rd0 [56]) superimposed on the crystal structure of p110γ with GSK2126458 bound (PDB code 3l08 [38]). PI3Kα^WT^ amino acids are displayed. The red sphere represents a water molecule included in the docking model of **5**, and the cyan sphere represents a water molecule observed in the crystal structure for GSK2126458. (**D**) BLI wavelength shift (λ) data for P2pY activated PI3Kα^WT^ (1 µM) membrane binding in the presence of GSK2126458 (GSK; 4 µM), **6** (4 µM), **7** (10 µM). Results are shown as the Mean ± SD; no. of biosensors used: DMSO *n* = 16; GSK *n* = 10; **6** *n* = 9; **7** *n* = 8.

We next sought to establish a pharmacophore explaining the antagonist effect of GSK2126458 on the PI3Kα^WT^ membrane interaction. We previously described a PI3K inhibitor series [37] that shared features with known PI3Kα inhibitors alpelisib [16] and A66 [37], differing only in the moieties that access the ATP site affinity pocket with one compound possessing a difluorophenyl-sulfonamide inspired by GSK2126458 (Supplemental Figure S3A–F). We investigated the intrinsic fluorescence properties of this series and considered only compounds **1**, **2**, **3**, and **5** suitable for use in the FRET assay (Supplemental Figure S3G). Of these, **5** showed the largest reduction in the FRET signal (Figure 5A), with the membrane interaction effect confirmed by BLI (Figure 5B). The BLI data for **4** compared with **5** indicated that the difluorophenyl-sulphonamide group was essential for antagonising the PI3Kα^WT^ membrane interaction (Figure 5B, Supplemental Figure S3A).

The difluorophenyl-sulphonamide in GSK2126458 was proposed to make an electrostatic interaction with Lys833 in PI3Kγ [38], a catalytically important amino acid [40] conserved across PI3Ks [41]. Superposition of a molecular docking model of **5** in the PI3Kα^WT^ ATP binding site onto the X-ray crystal structure of GSK2126458 bound p110γ (PDB: 3L08) suggested that the difluorophenyl-sulphonamide group in both compounds could make similar interactions (Figure 5C). Comparison of the binding models also showed that **5** and GSK2126458 could form a hydrogen bond with the backbone amide of Val851 in the linker region (Figure 5C) [37].

To understand if the Val851 interaction contributed to the effect of difluorophenyl-sulphonamide containing compounds on the PI3Kα^WT^ membrane interaction, we designed and synthesised **6**, an analogue of GSK2126458 where the central quinoline group was replaced by naphthalene, removing the hydrogen bonding capability (Supplemental Figure S4). We confirmed GSK2126458 as a potent inhibitor of PI3Kα [37,38] with an IC_50_ of 0.4 nM (Supplemental Table S1), then showed that removing the ligand's hydrogen bond acceptor atom decreased potency for PI3Kα^WT^ by >1000× and that the compound had similar potencies for other class I PI3K enzymes (Supplemental Table S3). BLI data showed that **6** retained the ability to decrease the P2pY-activated PI3Kα^WT^ membrane interaction as did the extremely weak difluorophenyl-sulphonamide containing inhibitor **7** [42] (Figure 5D, Supplemental Figure S4, Supplemental Table S3). From these data we considered that a minimal pharmacophore for affecting membrane binding includes a difluorophenyl-sulphonamide able to interact with the catalytic lysine, while the maximal effects require a hydrogen bond acceptor able to interact with Val851.

### GSK2126458 regulates the PI3Kα^WT^ membrane interaction through a mechanism that requires the PI3Kα^WT^ pY-activated state to be able to revert to the basal state

**Figure 6.**
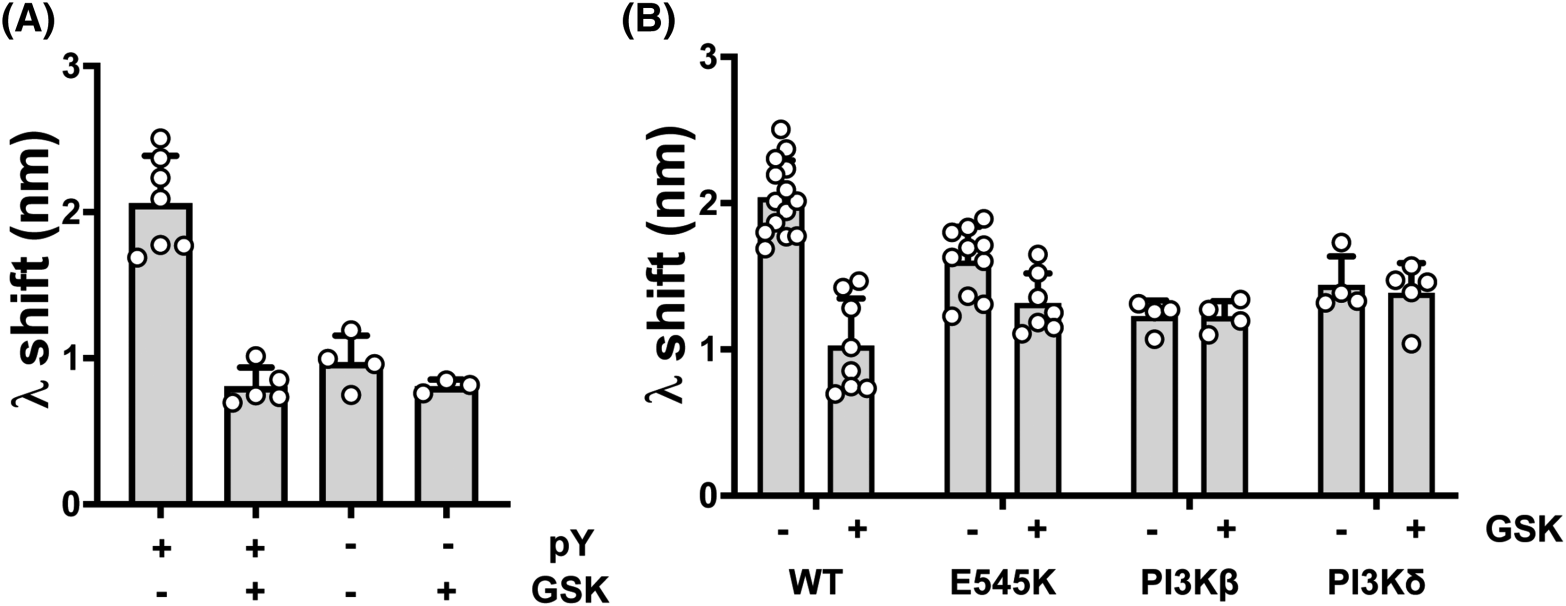
GSK2126458 only affects receptor activated PI3Kα^WT^. (**A**) Effect of P2pY (pY) activation on the PI3Kα^WT^ membrane binding BLI response in the presence and absence of GSK2126458 (GSK). The data are shown as Mean ± SD; PI3Kα^WT^ +pY *n* = 7; PI3Kα^WT^ +pY + GSK *n* = 5; PI3Kα^WT^ −pY *n* = 4; PI3Kα^WT^ −pY + GSK *n* = 3. (**B**) Effect of GSK on the BLI wavelength shift (λ) on membrane binding by PI3Kα^WT^, the PI3Kα^E545K^ oncogenic mutant, class IA PI3Kβ^WT^ and class IA PI3Kδ^WT^ enzymes. In all experiments inhibitors were tested at 4 µM with 1 µM of the different enzymes and membrane binding was followed by BLI for 300 s. The graph shows the wavelength shift in the presence or absence of compound as Mean ± SD; PI3Kα^WT^ +pY *n* = 14; PI3Kα^WT^ +pY + GSK *n* = 8; PI3Kα^E545K^ +pY *n* = 10; PI3Kα^E545K^ +pY + GSK *n* = 4; PI3Kβ^WT^ + pY *n* = 4; PI3Kβ^WT^ + pY + GSK *n* = 4; PI3Kδ^WT^ + pY *n* = 4; PI3Kβ^WT^ + pY + GSK *n* = 5.

Burke et al. [6] showed that release of the inhibitory interaction between the p85 nSH2 domain and p110α subunit in pY-peptide activated PI3Kα^WT^ increased the membrane interaction. Using BLI, we confirmed that P2pY-activation increased the membrane interaction (Figure 6A). We then showed that GSK2126458 only had an effect on the peptide-activated enzyme state compared with the basal state, reducing the membrane interaction to the same level as the basal enzyme state. However, ablating the inhibitory nSH2 interaction with an oncogenic E545K kinase domain mutation that is unaffected by peptide activation rendered the membrane interaction insensitive to regulation by GSK2126458 (Figure 6B). The membrane interaction of other p85α containing class IA enzymes PI3Kβ^WT^ and PI3Kδ^WT^ (Figure 6B) was also insensitive to GSK2126458, indicating that the membrane interaction mechanism regulated by GSK2126458 was specific to the pY-activated PI3Kα^WT^ enzyme. Finally, we demonstrated that the p85α cSH2 domain was not required for GSK2126458 to regulate the PI3Kα^WT^ membrane interaction using a PI3Kα enzyme containing p85α truncated after position 602 (Supplemental Figure S5).

### Oncogenic mutations occurring in the p110α ABD-RBD linker region are sensitive to regulation by GSK2126458

To understand if the regulatory mechanism used by GSK2126458 was restricted to PI3Kα^WT^ or if other oncogenic mutants were also sensitive to its effects, we studied the membrane interaction affinity and capacity for mutations with diverse and well described effects on autoinhibitory interactions in the p110α-p85α heterodimer [6] (Figure 1C).

Our data showed that the membrane interaction for mutations outside of the p110α kinase domain was sensitive to regulation by GSK2126458, and that for mutations within the kinase domain, regulation is location dependent. Regulation was clearly evident for the ABD-RBD linker region mutations G106V and G118D. The G106V mutation showed the largest decrease in capacity of all those tested, while its affinity was unaffected, similar to the wild type enzyme. GSK2126458 affected the G118D membrane interaction causing changes in both capacity and affinity, suggesting a change in the membrane interaction mechanism. The p110α C2 domain mutations N345K and C420R at the p110α-p85α iSH2 domain interface were also sensitive to GSK2126458, with effects on the membrane interaction capacity. Interaction capacity for the kinase domain C-terminal tail mutations M1043I and G1049R were also sensitive to GSK2126458, unlike the H1047R and helical domain E545K hotspot mutations (Supplemental Figure S6A–C).

### ATP analogues and ATP-site directed inhibitors have divergent effects on the PI3Kα^WT^ membrane interaction

**Figure 7.**
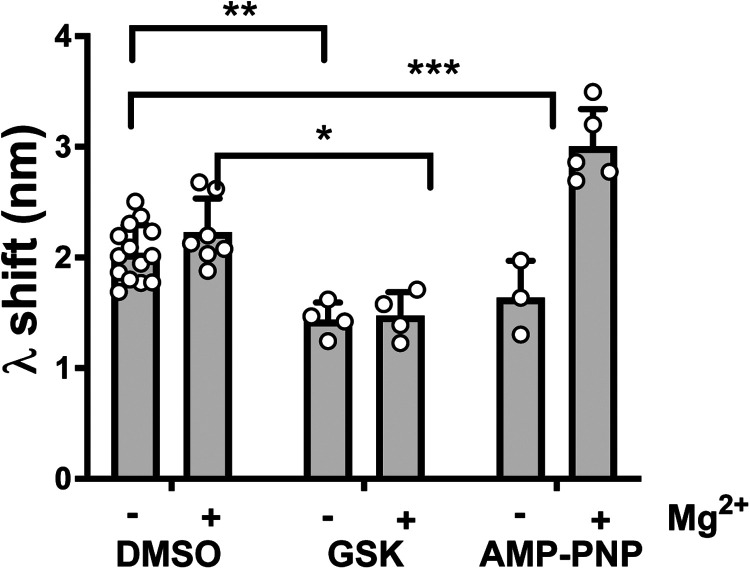
Nucleotide binding increases PI3Kα^WT^ membrane binding. Effect of GSK2126458 (GSK) and the non-hydrolysable ATP analogue AMP-PNP on the BLI wavelength shift (λ) for membrane binding by PI3Kα^WT^ in the presence and absence of 5 mM Mg^2+^. GSK was used at 4 µM with 1 µM of P2pY activated PI3Kα^WT^ and AMP-PNP was used at 2 mM. Membrane binding was followed by BLI for 300 s. The graph shows the wavelength shift in the presence or absence of compound as Mean ± SD; PI3Kα^WT^ DMSO −Mg^2+^ *n* = 14, DMSO + Mg^2+^ *n* = 7, GSK −Mg^2+^ *n* = 4, GSK+Mg^2+^ *n* = 4, AMP-PNP −Mg^2+^ *n* = 3, AMP-PNP +Mg^2+^ *n* = 5.

Based on our finding that the PI3Kα^WT^ membrane interaction can be negatively or positively regulated by ATP-site directed inhibitors, we explored whether these responses might mimic the effect of ATP substrate binding to PI3Kα^WT^. BLI data for the non-hydrolysable ATP analogue, AMP-PNP, in the presence of Mg^2+^ clearly showed that nucleotide substrate binding could promote the enzymes membrane interaction and that Mg^2+^ was important for the outcome (Figure 7). We then screened a range of inhibitor chemotypes (Supplemental Figure S7A) and discovered that apart from GSK2126458 no other inhibitors showed negative regulation of the PI3Kα^WT^ membrane interaction to a level that reached statistical significance (Supplemental Figure S7B). Notwithstanding this, the data for ZSTK474 was consistent with its FRET response (Figure 2B) that showed a decrease in the membrane interaction. Conversely, AS252424 showed some evidence of positive modulation (Supplemental Figure S7B), and the PI3Kβ sparing inhibitor GDC0032 significantly increased the PI3Kα^WT^ membrane interaction (Supplemental Figure S7C). Along with PIK-75, these data show that it is possible for catalytic site inhibitors to also have effects on membrane binding that work against the catalytic inhibition.

## Discussion

PI3Kα plays a central role in transducing signals from cell surface receptors into downstream signalling networks, with its enzyme activity and physical interactions tightly regulated by the plasticity of both the catalytic and regulatory subunits of the heterodimeric protein. Understanding how small molecule inhibitors of PI3Kα affect essential macromolecular interactions like membrane binding is necessary for a complete definition of the mechanism of action of ATP binding site inhibitors and the discovery of compounds with specific mechanisms of action. In this study we separated the PI3Kα membrane interaction from catalysis using FRET and BLI based biophysical membrane interaction assays. We showed that some ATP binding site directed inhibitors regulate the PI3Kα membrane interaction in its growth factor activated state, with some compounds mimicking the effect of nucleotide binding by promoting membrane interaction while others antagonise the enzyme's membrane interaction. Benzene-sulphonamide containing inhibitors represented by GSK2126458 form a new class of inhibitor with a dual mechanism of action that blocks ATP binding and acts as a full antagonist of the phosphopeptide-activated PI3Kα^WT^ membrane interaction. Antagonism of the membrane interaction by this class of compound is specific to PI3Kα^WT^ and is dependent on the enzyme being able to access its basal state in the presence of the compound. Our results answer the question about the effect of inhibitors on the PI3Kα membrane interaction by demonstrating that regulation of the membrane interaction is coupled to ATP binding site occupancy.

Our BLI data showed that the phosphotyrosine peptide activated PI3Kα^WT^ had enhanced membrane interaction, consistent with previous FRET and SPR assays [5,6]. PI3Kα activation by growth factor receptor mimicking phosphotyrosine containing peptides increases the plasticity of PI3Kα. Activation involves the release of intersubunit inhibitory constraints between the p85α nSH2 domain and the p110α helical domain, the p85α coiled coil region (iSH2 domain) and the p110α-C2 domain, as well as intrasubunit movement of the p110α ABD relative to other p110α domains. These changes are also promoted by oncogenic mutations [6]. Within the p110α subunit, conformational changes associated with activation also allow movement of the helical regulatory motif Kα8 and Kα11, these changes release an inhibitory constraint from the C-terminus helix in the kinase domain and potentially expose the WIF motif important for PI3Kα^WT^ membrane binding to better interact with the membrane [5,43].

Molecular dissection of the membrane interaction antagonism by GSK2126458 was performed using p110α oncogenic mutations that affect specific, well characterised, regulatory processes [6,43]. Together, these mutations showed that antagonism of the membrane interaction by GSK2126458 is dependent on the PI3Kα^WT^ enzyme adopting a native basal state that includes the inhibitory nSH2-p110α and C-terminus helix interactions. The nSH2 inhibitory effect results from binding to the p110α helical, kinase and C2 domains, stabilising the C2-iSH2 interaction and preventing the movement of the ABD relative to other p110α domains [43]. Ablation of the wild type nSH2-p110α helical domain interaction by the E545K mutation rendered the membrane interaction insensitive to drug regulation, suggesting that although PI3Kα^E545K^ mimics the P2pY-stimulated state, the difference in response compared with the P2pY-activated PI3Kα^WT^ enzyme is due to the inability of PI3Kα^E545K^ to be converted back into a non-P2pY stimulated state. It also shows that the remaining nSH2 interaction sites on the p110α protein are insufficient to create a native-like interface that can overcome the oncogenic change. Our data showed that the H1047R mutant membrane interaction was also not regulated by GSK2126458. The H1047R mutation was recently shown to affect the p110α regulatory motif, removing inhibitory interactions between the C-terminus, Kα11 and the activation loop, potentially reorienting C-terminus membrane binding motifs into a productive binding conformation [43]. The inability of GSK2126458 to modulate membrane binding for the mutant enzyme demonstrates that the H1047R substitution prevents the enzyme from adopting a native-like state where inhibitory interactions between the regulatory motif and the activation loop are re-instated, supporting a model where steric effects of the substitution are not accommodated at the Kα11 activation-loop interface [43]. Our observation that the membrane interaction for mutations in regions of the regulatory motif on either side of H1047 are sensitive to GSK2126458 isolate the interaction between the Kα11 and the activation loop as an important determinant for modulation by GSK2126458.

Mutations that increase conformational freedom at the iSH2-p110α interface (N345K, C420R), or at the ADB-RBD linker (G106V, G118D) [6] retained sensitivity to the effect of GSK2126458 on membrane interaction. These outcomes are consistent with observations that mutations at the iSH2-p110α and ADB-RDB interfaces remain responsive to activation by release of the nSH2 interaction [6]. Even though these mutations affect specific PI3Kα activation steps by modifying different interdomain interfaces [6], our data shows that the effect of these changes are not sufficient to overcome the drug induced switch to a basal state. Based on these data, we hypothesise that GSK2126458 selects for p110α conformations that promote assembly of the p85α-p110α complex including the nSH2-p110α interaction, shifting the peptide activated enzyme to a basal-like state that is less responsive to phosphotyrosine containing peptide than the enzyme with no inhibitor bound.

Our essential structure activity relationship study showed that the negative regulatory effects of the benzene sulfonamide containing inhibitors were most effective when the ligands could interact with both the linker region found at the base of ATP binding site, and a conserved Lys located in the N lobe of the kinase domain [37,38]. These polar interactions are also shared with the ATP substrate, yet a non-hydrolysable ATP analogue had an opposite, positive, regulatory effect on the membrane interaction. When we compared the ligands crystallised in the p110γ protein [38,44] the differences in contacts made with the activation loop suggest that conformational selection of this region may contribute to the regulatory mechanism.

Communication between the activation loop that contains PIP_2_ lipid substrate recognition sites [5,8], the p110α regulatory motif and iSH2 and nSH2 domains of p85α provide a compelling mechanism for coupling inhibitor binding to membrane interaction [9,43]. Allosteric communication between the nSH2 domain and activation loop was predicted by molecular dynamics simulations. With physiologically relevant ligands bound, the simulations indicated that the nSH2 domain influences the active conformation of activation loop amino acids [45]. It may be anticipated that different inhibitor chemotypes select specific protein conformations that have positive, negative or neutral effects on the membrane interaction via the regulatory motif and helical domain-nSH2 interactions. Our data for PIK-75 and GDC-0032 support this hypothesis and show that different inhibitor chemotypes can mimic ATP and have positive regulatory effects.

Conformational selection within the kinase domain by ATP directed inhibitors involves other ATP binding site features along with the activation loop. Hydrogen deuterium exchange mass spectrometry [46] showed that GSK2126458 binding affects the PI3Kα ATP binding site conformation around the P-loop residues 770 to 780 in the p110α protein. Inhibitor induced changes in P-loop conformation were also identified in the wortmannin bound PI3Kα X-ray crystal structure [14]. Selection of specific active site conformations upon inhibitor binding is also well established in the p110γ and PI3Kδ enzymes as a mechanism for achieving selectivity [25,26].

Modulation of protein conformation was also proposed as part of the mechanism for new allosteric PI3Kα ligands, including the allosteric PI3Kα activator 1938 [27], and mutant selective allosteric inhibitors STX-478 [47] and RLY-2608 [48]. Activator binding outside of the ATP binding site was able to capture a protein conformation that increased the p110α membrane interaction, and this was related to global conformational changes that included the activation loop PIP_2_ binding sites. Similarly, the new allosteric inhibitors bind in cryptic sites that involve substantial re-arrangement of the activation loop [47–49]. Some of these binding sites are far away from the oncogenic mutant locations and more accessible to drug binding in kinase and helical domain mutant proteins compared with the wild type enzyme.

Our demonstration that ATP substrate analogue binding also increases the membrane interaction identifies a new, distinct mechanism to support PI3Kα-membrane interactions in addition to the release of p85α inhibitory constraints, release of the C-terminus WIF motif [43] and, partnering with Ras [7]. It is tempting to speculate that ATP binding may prime the enzyme for the membrane interaction.

In summary, we have shown that molecules occupying the ATP binding site of PI3Kα can regulate its membrane interaction and this has potential to influence the design of new ATP competitive inhibitors for this enzyme. We provide evidence that a class of benzene-sulfonamide containing ATP-competitive inhibitors represented by GSK2126458 have a dual mechanism, including inhibition of the PI3Kα membrane interaction. The membrane interaction effect is mediated by conformational changes in the p110α protein that are important for binding to the p85α regulatory protein and are consistent with the p110α occupying a basal state that is insensitive to phospho-peptide activation. Improved membrane interaction can also be encoded into small molecule inhibitors of different chemotypes as shown by our data with GDC0032 and PIK-75 increasing membrane association and this mimics a regulatory effect of ATP. We anticipate that deeper understanding of the conformational changes induced around the membrane interface could be exploited to develop new PI3Kα selective compounds with new mechanisms of action that can be used to gain more insight into non-catalytic functions of membrane localised and cytosolic PI3Kα. Finally, knowledge of an activator site that affects the activation loop conformation and the communication between the activation loop and PI3Kα regulatory features lead to the hypothesis that the activation loop is a central regulator of PI3Kα membrane interaction competent conformations.

## Materials and methods

### Compounds

GSK2126458 (SYNthesis and MedChemExpress), BEZ235 (synthesised in-house [50]) ZSTK474 (synthesised in-house [51], compound 16 (synthesised in-house following the method in [52]), CZC24832 (Selleckchem), BYL-719, GDC0941, GDC0032, PI-103 were purchased from MedChemExpress, AS252424 [53,54], PIK-75 [55], Wortmannin (Sigma–Aldrich).

### PI3Kα protein preparation

Proteins were purified using three separate protocols, Protocol A is as previously described [6], Protocol B followed the method in Ref. [37], and Protocol C followed a modified form of the method in Ref [37]. Sf9 cells (Life Technologies; Carlsbad, CA, U.S.A.) were infected with recombinant baculovirus containing coding sequences for the full length p110α/p85α (PI3Kα^WT^), p110β/p85α (PI3Kβ^WT^), p110δ/p85α (PI3Kδ^WT^) or p110γ subunits. Cell cultures were centrifuged at 666 × ***g*** and pellets were resuspended in an equal volume of cell pellet in 1x TBS (Tris-buffered saline; 25 mM Tris pH 8.0 and 137 mM NaCl). The pellets were then flash frozen in liquid nitrogen and stored at −20°C. The frozen cell pellets were then lysed and lysates were clarified by centrifugation at 20 000 × ***g***, the supernatant was removed and adjusted to 5% v/v glycerol, 150 mM NaCl, 7.5 mM imidazole and 20 µg/ml RNAseA (Roche) and then passed through a 0.45 µm filter before loading onto a Talon-Co^2+^ resin column (Clontech; Mountain View, CA, U.S.A.). Protein was eluted from the column with 25 mM Tris pH 8.0, 150 mM NaCl, 150 mM imidazole and 5% glycerol and then dialysed overnight against a buffer containing 50 mM Tris pH 8.0, 100 mM NaCl, 1 mM DTT and 5% glycerol. The dialysed protein was then desalted to 50 mM Tris pH 8.0, 40 mM NaCl, 1 mM DTT and 5% glycerol using a Pharmacia Hi-trap desalting column (GE Healthcare; Little Chalfont, U.K.) and applied to a MonoQ column (GE Healthcare) equilibrated in the same buffer. The protein was fractionated over a NaCl gradient from 40 to 500 mM NaCl in the presence of 50 mM Tris pH 8.0, 1 mM DTT and 5% glycerol over 20 min with 1 ml fractions collected. The protein typically eluted at ∼175 mM NaCl. Eluted protein was flash frozen in liquid nitrogen and stored at −80°C until used. Where protein was purified in HEPES, the same protocol was used with the substitution of Tris pH 8.0 for HEPES, pH 7.5.

### PI3Kα mutant preparation

Single point mutants G106V, G118D, N345K, C420R and M1043I were generated using non-overlapping antiparallel 5′ phosphorylated primers with the mutating nucleotides positioned at the extreme 5′ position of the forward primer, indicated by the italicised sequence in the primer list (Supplemental Table S5). Forward and reverse primers for each mutant were used in whole plasmid PCR using the pDUAL™ vector (Invitrogen) with the full length regulatory p85α subunit cloned in the p10 multiple cloning site and the full length catalytic p110α subunit cloned in the pPOL multiple cloning site of the vector. PCR reactions were performed in a 30 µl reaction volume containing 1 x polymerase buffer with 0.5 µM of each primer, 1.25 mM dNTPs (Invitrogen), 10 ng of vector template and 1.25 units of iProof High Fidelity Polymerase (Bio-Rad). Typical PCR conditions used an initial denaturation at 95°C for 5 min followed by 2 cycles of incubation at 95°C for 1 min, then 60°C for 15 s, then 72°C for 8 min; followed by 2 cycles of incubation at 95°C for 1 min, then 58°C for 15 s, then 72°C for 8 min; followed by 25 cycles of incubation at 95°C for 1 min, then 56°C for 15 s, then 72°C for 8 min; and finally terminated with an additional incubation at 72°C for 10 min. PCR product DNA was then precipitated by the addition of 5 µl 3 M sodium acetate pH 5.2 then 120 µl absolute ethanol, mixed by inversion and incubated at −20°C for 60 min. The DNA was subsequently pelleted by centrifugation at 14 000 × ***g*** for 45 min, the supernatant discarded and the pellet washed with ice cold 70% ethanol, then air dried and resuspended in 20 µl 10/1 TE buffer (10 mM Tris pH 8.0, 1 mM diNa^+^ EDTA) and the concentration measured by UV spectroscopy. Up to one microgram of PCR product was self-ligated in a 10 µl volume with 0.5 units of DpnI (New England Biolabs) to remove parental template DNA and 100 units of T4 DNA ligase (New England Biolabs) overnight at room temperature. Ligation products were transformed into *Escherichia coli* DH5α and grown overnight at 37°C on LB agar supplemented with 75 µg/ml ampicillin. Individual colonies were then selected and grown overnight at 37°C in 2.5 ml of LB supplemented with 75 µg/ml ampicillin. Plasmids were extracted from individual colonies and mutations confirmed by Sanger sequencing. All sequencing was performed by the Genomics Centre, Auckland Science Analytical Services, The University of Auckland, Auckland, New Zealand.

### Homogenous time-resolved fluorescence lipid kinase IC_50_ determination

IC_50_ values were determined using the PI3K homogenous time-resolved fluorescence assay (Merck Millipore, #33-016) following the manufacturer's instructions as previously described [37]. PI3K proteins were used at the following concentrations: 65 ng/ml for PI3Kα^WT^, 30 ng/ml for PI3Kα^E545K^ mutant, 65 ng/ml for PI3Kα^H1047R^ mutant, 230 ng/ml for PI3Kβ^WT^, 30 ng/ml for PI3Kδ^WT^, 300 ng/ml for p110γ. Inhibitors were prepared and serially diluted in 100% DMSO, and the final DMSO concentration used in the reaction was 2.5%. Data was graphed and analysed with Graphpad Prism 6, nonlinear regression with log(inhibitor) vs. response and constraints set at 0% and 100%.

### Liposome preparation

Lipid vesicles were prepared as previously described with some modifications [6]. Lipid components dissolved in chloroform were mixed together and then the solvent was evaporated under a stream of nitrogen gas. The remaining lipid film was dried under a vacuum for 2 h, then resuspended in 2 ml of either Buffer A (20 mM Tris pH 7.5, 100 mM KCl and 1 mM EGTA) or Buffer B (20 mM HEPES pH 7.5, 100 mM KCl and 1 mM EGTA) followed by continuous vortexing for 3 min, then sonication in a water bath for 2 min at room temperature. The lipid solution was then subjected to 11 freeze-thaw cycles by snap freezing in liquid nitrogen followed by thawing in a 42°C water bath. Liposomes were then created by extruding 11 times through a 100-nm filter, and frozen in liquid nitrogen and stored at −80°C. They were thawed at room temperature before use.

### Forester resonance energy transfer membrane binding assay

Liposomes were prepared with PIP_2:_ phosphatidylserine (PS): phosphatidlyethanol (PE): phosphatidylcholine (PC): Cholesterol (C): Sphingomyelin (S): Dansyl PS (Avanti polar lipids) in the percentage of 5: 20: 35: 15: 10: 5: 10. Inhibitors were solubilised in 100% DMSO, and were diluted to 10% DMSO with Buffer C (30 mM HEPES pH 7.5, 50 mM NaCl), before being serially diluted to desired concentrations in 30 mM HEPES pH 7.5, 50 mM NaCl with 10% DMSO. PI3Kα^WT^ was pre-incubated with 10 μM phosphopeptide (pY) for 10 min, but pY was not added to the PI3Kα^H1047R^ mutant. Two different peptides were used in this study, P1pY (ESDGG(pY)MDMSKDESID(pY)VPMLDMKGDIKYADIE) and P2pY (CSDGG(pY)MDMSKDESVD(pY)VPMLD). The concentration of enzyme for the FRET assay was determined using full length PI3Kα^WT^ and PI3Kα^H1047R^ up to 1 μM to determine the midpoint of the FRET binding signal, and final concentrations of 0.5 and 0.2 μM were selected. Compounds were used at 5-fold the final concentration and 2 μl was added to 5 μl of enzyme in a 384-well plate, the plate was sealed and the mixture was allowed to equilibrate at room temperature for 10 min with shaking at 450 rpm. Liposomes were then added to the mixture to give a final liposome concentration of 50 μg/ml and a final reaction volume of 10 μl, and the plate was incubated at room temperature for 10 min with shaking at 450 rpm. The FRET signal was detected using a PHERAStar HTS microplate reader (BMG Labtech) with an optic module that selected excitation at 280 nm and emission at 350 and 520 nm. The FRET signal was measured as *I*-*I*_0_ (intensity at 520 nm for protein and liposome solution — intensity at 520 nm for liposome only solution). In experiments involving compounds, *I*-*I*_0_ was also determined for the compound only controls as intensity at 520 nm for the compound — intensity at 520 nm for liposome only solution). The compound only *I*-*I*_0_ was then subtracted from the *I*-*I*_0_ of the protein-compound complex. The FRET signals for each compound concentration were converted to a percentage of the FRET signal for the lowest concentration of inhibitor. Data were then graphed and analysed with GraphPad Prism 6.

### BLI membrane binding assay

BLI experiments were carried out with a BLItz® System (ForteBIO) using aminopropylsilane (APS) biosensors. The APS biosensors (ForteBIO #18-5045) were hydrated for 15–20 min in either Tris or HEPES liposome buffer. The program option used was Advanced Kinetics. Liposome membrane preparation was performed using a 0.5 mg/ml liposome solution of PIP_2:_ PS: PE: PC: C: S (Avanti polar lipids) in the mass percentages of 5: 20: 45: 15: 10: 5. Liposomes without PIP_2_ were prepared as PS: PE: PC: C: S in the percentage of 20: 50: 15: 10: 5. Liposomes without PS were prepared as PIP_2:_ PE: PC: C: S in the percentage of 5: 65: 15: 10: 5. APS tips were soaked in liposome solution using a 4 µl sample holder, and binding was recorded for 300 s after a 30 s baseline recording, followed by 120 s dissociation in Buffer A. The liposome bound biosensor tips were blocked with 2 mg/ml Chicken egg white albumin (Sigma #A7642) dissolved in Buffer A using a sample holder for 300 s, this was followed by 120 s dissociation in Buffer A. Full length PI3Kα^WT^ or PI3Kα^H1047R^ was diluted with Buffer D (50 mM Tris pH 8, 140 mM NaCl, 5% glycerol (v/v), 1 mM DTT). P2pY and inhibitor, or DMSO only were also added to achieve a final protein concentration of 200 μg/ml, a pY concentration of 10 μM, and total DMSO content of 3%. The mixture was incubated at room temperature for 10 min prior to the protein association experiments. The membrane coated biosensors were first dipped in Buffer D containing 3% DMSO for 30 s to set an initial baseline, and transferred to the protein in the presence or absence of inhibitor. Protein binding was followed for 300 s, and then the biosensors were dipped in Buffer D with 3% DMSO again for 300 s for protein dissociation. For concentration response experiments with and without GSK2126458 PI3Kα^WT, E545K, G1049R, N345K, G106V^ used the points 0.125, 0.25, 0.5, 1, 2, 3 µM, and PI3Kα^H1047R^ used 0.125, 0.25, 0.5, 1, 2, 4 µM. PI3Kα^C420R^ used 0.25, 0.5, 1, 1.9 µM, except when GSK2126458 was included where 0.5, 1 and 1.9 µM were used. PI3Kα^G118D^ used 0.125, 0.25, 0.5 1, 2 and 3 µM, except when GSK2126458 was included where the points 0.5, 1, 2 and 3 µM were used. PI3Kα^M1043I^ used 0.125, 0.25, 0.5, 1 and 1.57 µM. The results of liposome loading and protein association were then analysed and graphed using GraphPad Prism 6.

## Data Availability

The findings of this study are supported by the data within the article and its Supplementary Materials.

## Open Access

Open access for this article was enabled by the participation of University Of Auckland in an all-inclusive *Read & Publish* agreement with Portland Press and the Biochemical Society under a transformative agreement with CAUL.

## Abbreviations

APS: aminopropylsilane
BLI: biolayer interferometry
C: Cholesterol
PC: phosphatidylcholine
PE: phosphatidylethanolamine
PS: phosphatidylserine
S: sphingomyelin
