# Supplementary material for: ATP-competitive inhibitors of PI3K enzymes demonstrate an isoform selective dual action by controlling membrane binding
Source: Biochem J. 2024 Nov 28;481(23):1787–802. doi: 10.1042/BCJ20240479 (PMC7617104; doi:10.1042/BCJ20240479)
Supplement: Supplementary Material [file BCJ-481-1787-s1.pdf]

ATP-competitive inhibitors of PI3K enzymes demonstrate an isoform selective dual action by controlling membrane binding.

Grace Q. Gong[1,2], Glenn R. Masson[3], Woo-Jeong Lee[1,2], James M. J. Dickson[2,4], Jackie D. Kendall[2,5], Manoj K. Rathinaswamy[6], Christina M. Buchanan[1,2], Martin Middleditch[4], Brady M. Owen[1], Julie A. Spicer[2,5], Gordon W. Rewcastle[2,5], William A. Denny[2,5], John E. Burke[6], Peter R. Shepherd[1,2,5], Roger L. Williams[3], Jack U. Flanagan[2,5,7*]

[1]Department of Molecular Medicine, The University of Auckland, Auckland, New Zealand

[2]Maurice Wilkins Centre for Molecular Biodiscovery, The University of Auckland, Auckland, New Zealand

[3]MRC Laboratory of Molecular Biology, Francis Crick Avenue, Cambridge CB20QH, UK

[4]School of Biological Sciences, The University of Auckland, Auckland, New Zealand

[5]Auckland Cancer Society Research Centre, The University of Auckland, Auckland, New Zealand

[6]Department of Biochemistry and Microbiology, University of Victoria, Victoria, British Columbia, V8W 2Y2 Canada

[7]Department of Pharmacology and Clinical Pharmacology, The University of Auckland, Auckland, New Zealand

* To whom correspondence should be addressed.

Jack U. Flanagan: j.flanagan@auckland.ac.nz

Key words: lipid kinase, PI3K, PI 3-kinase, *PIK3CA*, membrane binding, small molecule, conformational change, inhibitor, biolayer interferometry, membrane

Table S1. IC_{50} values for known ATP-site directed PI3K inhibitors.

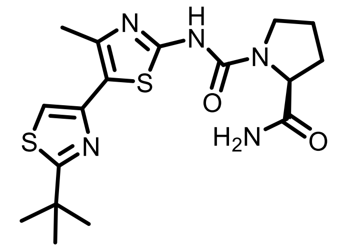

A66

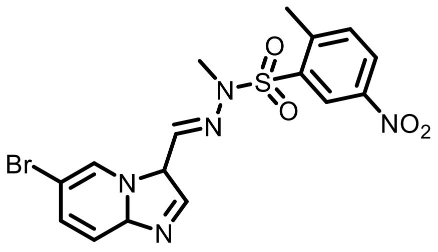

PIK-75

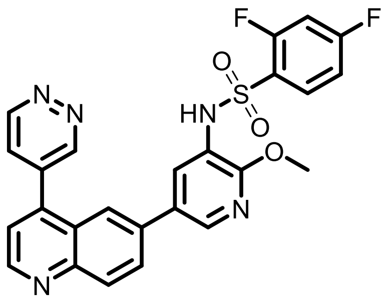

GSK2126458

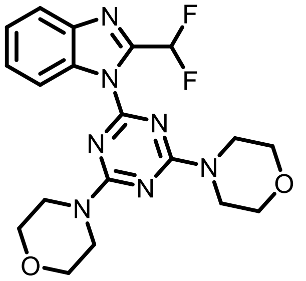

ZSTK474

Inhibitor	IC_{50} (nM)				
	PI3Kα^{WT}	PI3Kα^{H1047R}	PI3Kβ^{WT}	PI3Kδ^{WT}	p110γ^{WT}
#A66	44 ± 22	36 ± 22	>13000	2700 ±700	2700 ± 1500
#PIK-75	6 ± 2	2 ± 0.1	50 ± 18	170 ± 60	43 ± 21
#GSK2126458	1 ± 0.4	1 ± 0.1	2 ± 1	1 ± 0.5	1 ± 0.1
~GSK2126458	0.4				
ZSTK-474	15 ± 10	11 ± 6	7 ± 4	2 ± 1	8 ± 1
BYL-719	[a]32 , 9		[b]12000	[a]534 , 995	[b]746
GDC0032	[a]0.7 , 1.3		[a]64 , 113	[a]0.7 , 1.1	[a]4.6 , 3.2

Data from S1

~ Data from this study n=3.

a N=2 individual data shown

b N=1 individual data points shown

Table S2. IC_{50} values for ATP-site directed inhibitors in the FRET membrane binding assay.

Inhibitor	pIC_{50} (M) ± SE<br>$PI3K\alpha^{WT}$	IC_{50} (nM)<br>$PI3K\alpha^{WT}$
A66	6.64 ± 0.14	238
PIK-75	N.D.	N.D.
GSK2126548	6.56 ± 0.06	273
ZSTK-474	6.62 ± 0.04	240

Data were modelled using the variable slope method with four parameters in Graphpad Prism 6 and are the mean of 3 independent experiments. N.D. not determined. Protein was made using protocol A.

Figure S1. Representative BLI sensograms for the different protein concentrations used in binding parameter determination. (A) PI3Kα^{WT} with DMSO, (B) PI3Kα^{WT} with GSK2126458 at a 1:4 ratio (C) PI3Kα^{H1047R} with DMSO (d) PI3Kα^{H1047R} with GSK2126458 at a 1:4 ratio. All enzymes were activated with P2pY. Data are representative association sensograms from the set of tips collected at each concentration combined with the dissociation sensogram.

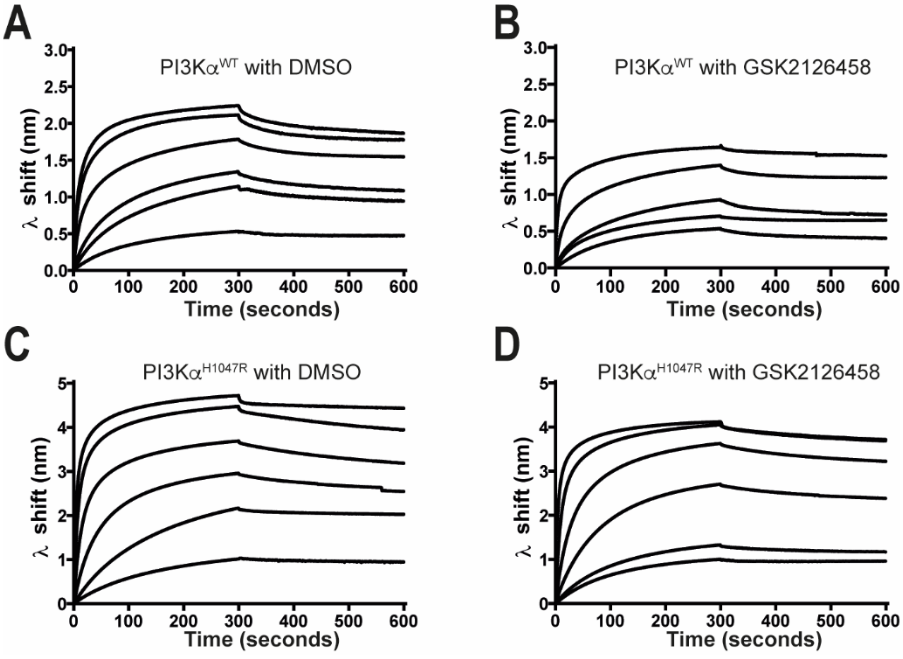

Figure S2. Effect of GSK2126458 and PIK-75 on PI3Kα^{WT} and PI3Kα^{H1047R} membrane binding parameters for liposomes with different anionic lipid compositions immobilised on a BLI biosensor.

(A) Comparison of PI3Kα^{WT} binding to liposomes with 0% PIP_2 in the presence and absence of GSK2126458. (B) Comparison of PI3Kα^{WT} binding to liposomes with 5% PIP_2 and 20% PS in the presence and absence of PIK-75; (C) Comparison of PI3Kα^{WT} binding to liposomes with 0% PIP_2 in the presence and absence of PIK-75; (D) Comparison of PI3Kα^{H1047R} binding to liposomes with 0% PIP_2 in the presence and absence of GSK2126458; (E) Comparison of PI3Kα^{H1047R} binding to liposomes with 0% PS in the presence and absence of GSK2126458; (F) Comparison of PI3Kα^{H1047R} binding to liposomes with 5% PIP_2 and 20% PS in the presence and absence of PIK-75; (G) Comparison of PI3Kα^{H1047R} binding to liposomes with 0% PIP_2 in the presence and absence of PIK-75; (H) Comparison of PI3Kα^{H1047R} binding to liposomes with 0% PS in the presence and absence of PIK-75. The DMSO control is represented as the solid line, and the PI3Kα-drug treatment in shown as a dashed line. Data is shown as Mean ± SEM (no. of biosensors ≥3). The data was modelled with Prism using a one site-specific binding model. The protein to inhibitor ratio was maintained at 1:4 for both GSK2126458 and PIK-75 at each PI3Kα concentration. All PI3Kα enzymes were activated with P2yP.

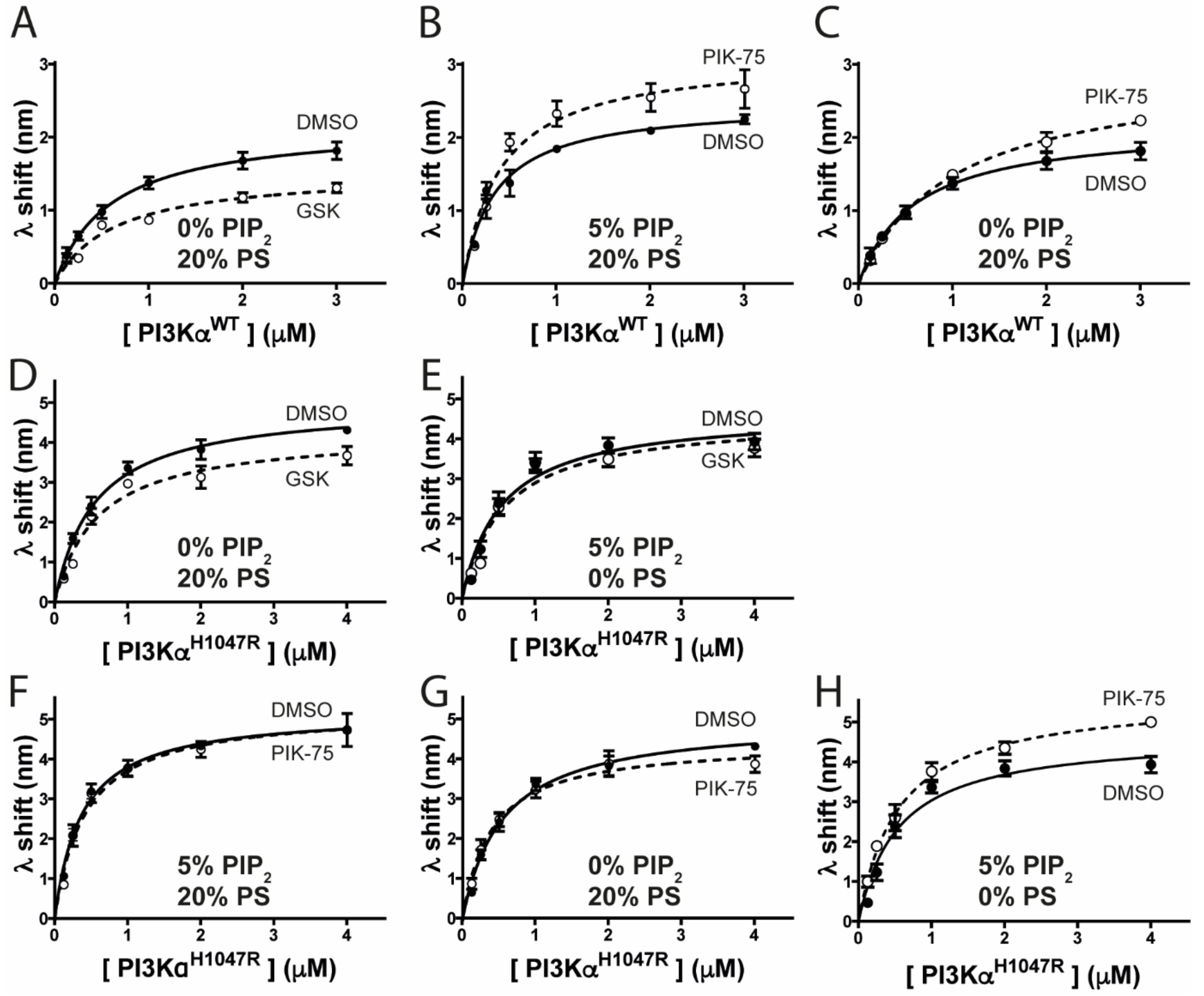

Figure S3. ATP-competitive inhibitor series, their PI3Kα molecular docking models and fluorescence properties. (A) Schemes of compounds **1-5** with the variable region shown in blue along with sulfonamide common to **5** and GSK2126458. (B)-(F) Molecular docking models of compounds **1-5** in human $PI3K\alpha^{WT}$ active site (PDB code 2RD0). The differences between the compounds are coloured cyan. (B) **1** positions a bromine atom in the affinity pocket. (C) **2** positions a 3-pyridyl group in the affinity pocket that could interact with a water molecule. (D) **3** contributes an NH_2-substituted 3-pyridyl group that is predicted to interact with the side chains of D933. (E) **4** contributed an OMe-substituted 3-pyridyl group and is predicted to interact with the side chain of K802 and a water molecule. (F) **5** is predicted to make an ionic interaction with K802 in addition to the interactions predicted for the OMe-substituted 3-pyridyl group of **4**. **5** also explores outside the affinity pocket with its difluorophenyl sulphonamide unit. (G) FRET analysis of compounds **1 - 5** in the presence of dansyl-labelled liposomes. Compounds were tested at 0.1 µM (black bars) and 10 µM (pink bars). The compound only $I-I_0$ was determined from I, the signal at 520 nm and I_0 the background fluorescence of dansyl labelled liposomes only. The experiment was done once with 4 replicates per inhibitor and the data is shown as Mean ± SD.

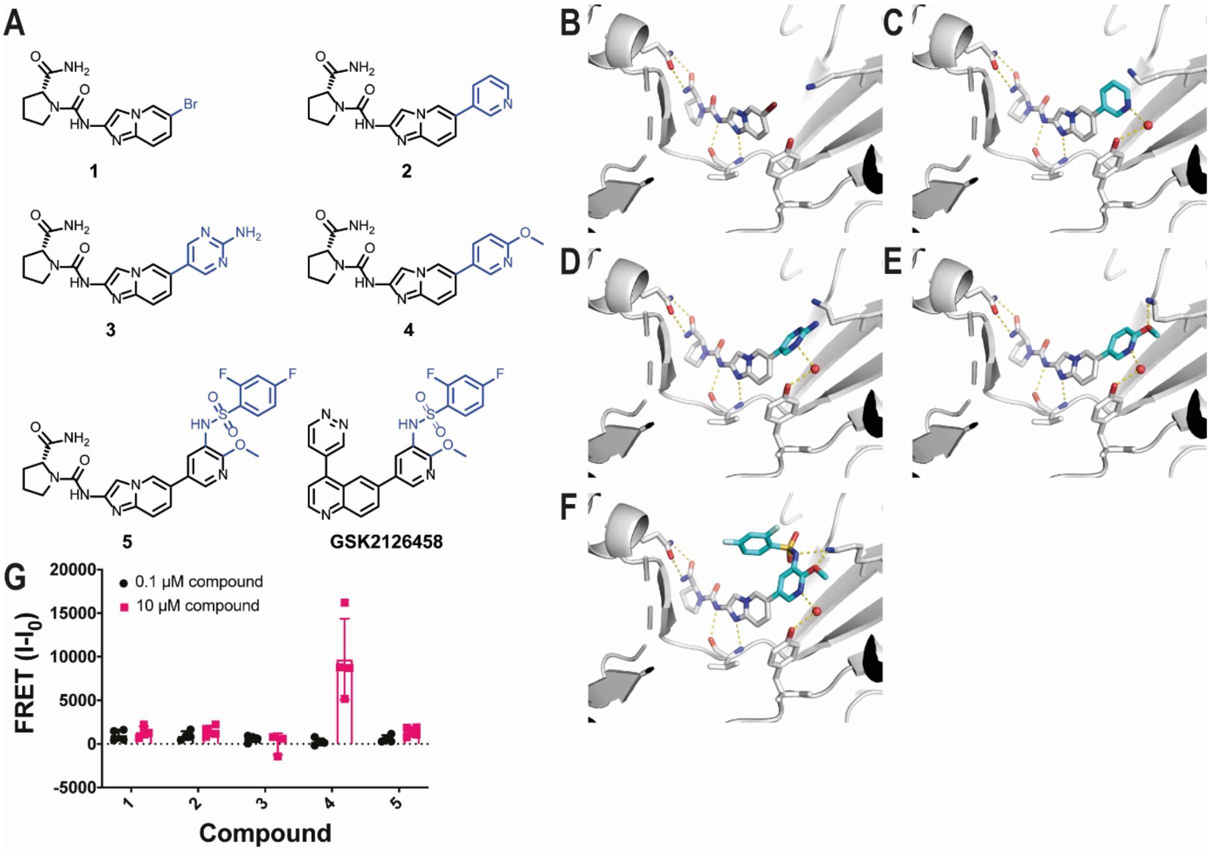

Figure S4. Design strategies for probing polar interactions between GSK2126458 and the PI3Kα ATP binding site. (Left) Illustration of GSK2126458 and the amino acid interactions investigated in this study. (Right) Illustration of how compounds **6** and **7** are predicted to explore the role of a hydrogen bond between GSK2126458 and the linker region Val851 backbone amide yet maintain the capacity to interact with Lys802 using a sulfonamide.

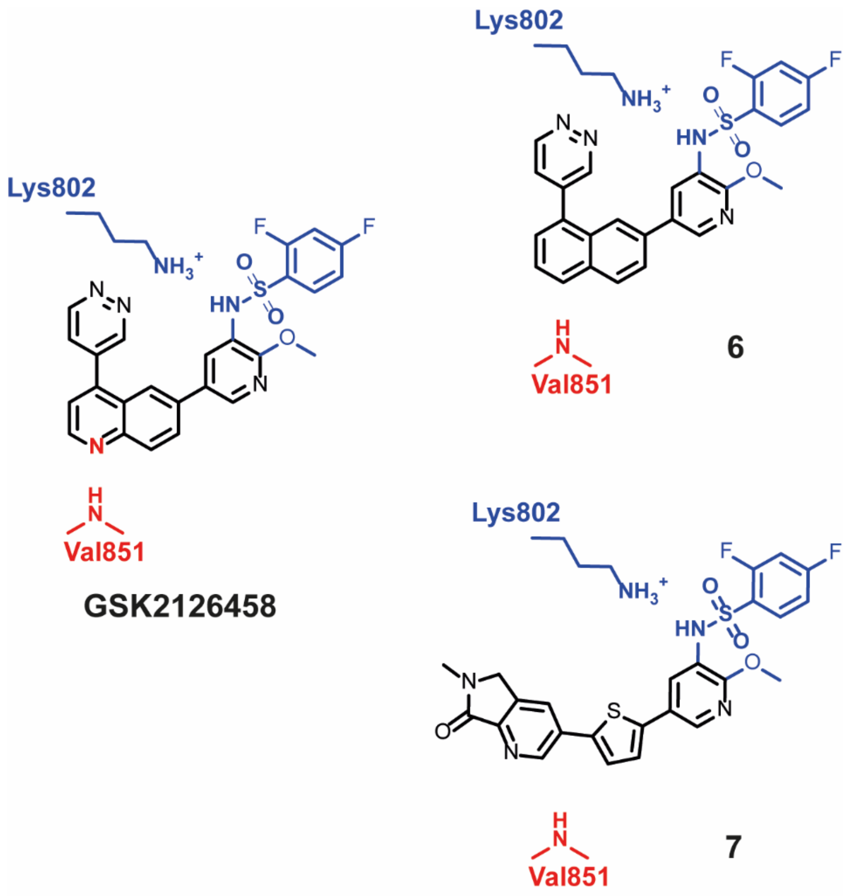

Figure S5. GSK2126458 affects the membrane binding of PI3Kα^{WT} with p85α truncated at position 602. Comparison of PI3Kα^{WT} binding to liposomes in the presence and absence of GSK2126458. The PI3Kα protein was produced using the protocol described in S2. The PI3Kα to GSK2126458 ratio was maintained at 1:4, and the enzyme was activated with P2yP. Data is shown as Mean ± SD. n=13 biosensors. Unpaired T-test, p value 0.008.

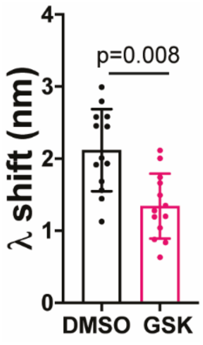

Table S3. IC_{50} data generated by HTRF assay using PIP_2 as the substrate.

Compound	IC_{50} (μM)		
	PI3Kα	PI3Kβ	PI3Kδ
No ATP site linker interaction			
**6**	0.46 ± 0.17	1.37 ± 0.23	0.15 ± 0.02
**7**	16.8 ± 9.1	242 ± 308	8.6 ± 2.1

Data shown as Mean ± SD. (n=2)

Enzymes were used at $PI3K\alpha^{WT}$ 40 ng/ml, $PI3K\beta^{WT}$ as either 400 ng/ml or 300 ng/ml, and $PI3K\delta^{WT}$ was 65 ng/ml. Plates were read on a PHERAStar HTS microplate reader (BMG Labtech).

Figure S6. GSK2126458 has oncogenic mutant PI3Kα dependent effects on membrane binding parameters. (A) Concentration response curves for PI3Kα oncogenic mutant proteins. Data is shown as the Mean ± SEM (number of biosensors for each data point is n≥3). A one site-specific binding model was used to fit the data. The protein to GSK2126458 ratio was maintained at 1:4 for each PI3Kα concentration and the PI3Kα protein was activated with P2yP. (B) Comparison of Kd (Black) and Bmax (Pink) values for each mutant retrieved from the concentration response data. (C) Difference between the Bmax values retrieved with the DMSO vehicle compared to GSK2126458.

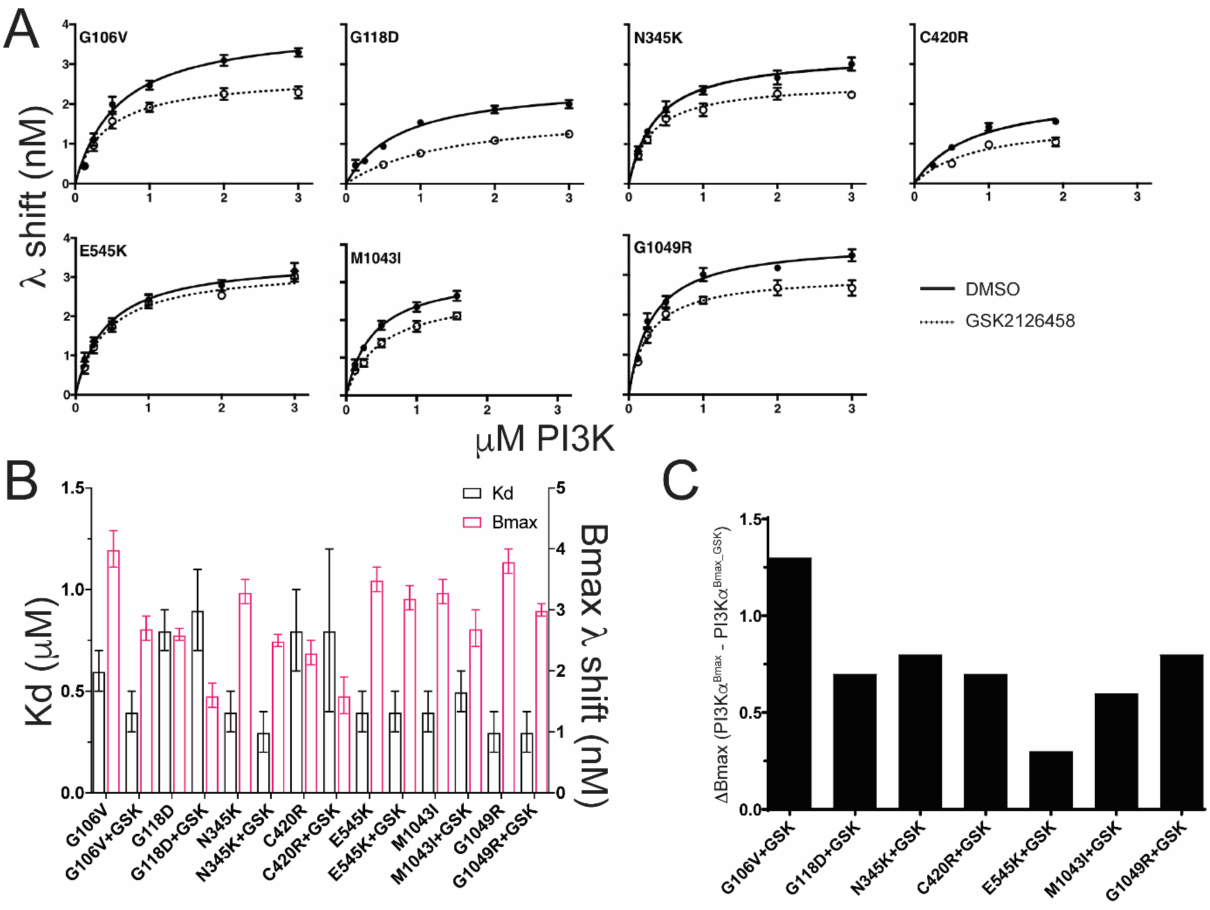

Figure S7. Expanded set of ATP site directed inhibitors that affect PI3Kα^{WT} membrane binding.

(A) Chemical structures for the compounds tested. (B) Effect of different types of chemistry on PI3Kα^{WT} membrane binding. PI3Kα^{WT} was kept at 1 μM with inhibitors used at different concentrations. 4 μM inhibitor included BYL-719 (n=2), GDC-0941 (n=3), GSK2126458 (GSK) (n=8); 10 μM inhibitor included A66 (n=3), PI-103 (n=5), BEZ-235 (BEZ) (n=3), compound 16 (Cmpd16; ref S3) (n=3), ZSTK474 (ZSTK) (n=3), AS252424 (n=6), CZC24832 (CZC) (n=4), staurosporin (Stauro) (n=2), wortmannin (Wort) (n=2). (C) Effect of GDC-0032 on PI3Kα^{WT} membrane binding. PI3Kα^{WT} was used at 1 μM, GSK2126458 (GSK) was used at 4 μM, PIK-75 at 4 μM and GDC-0032 at 10 μM.

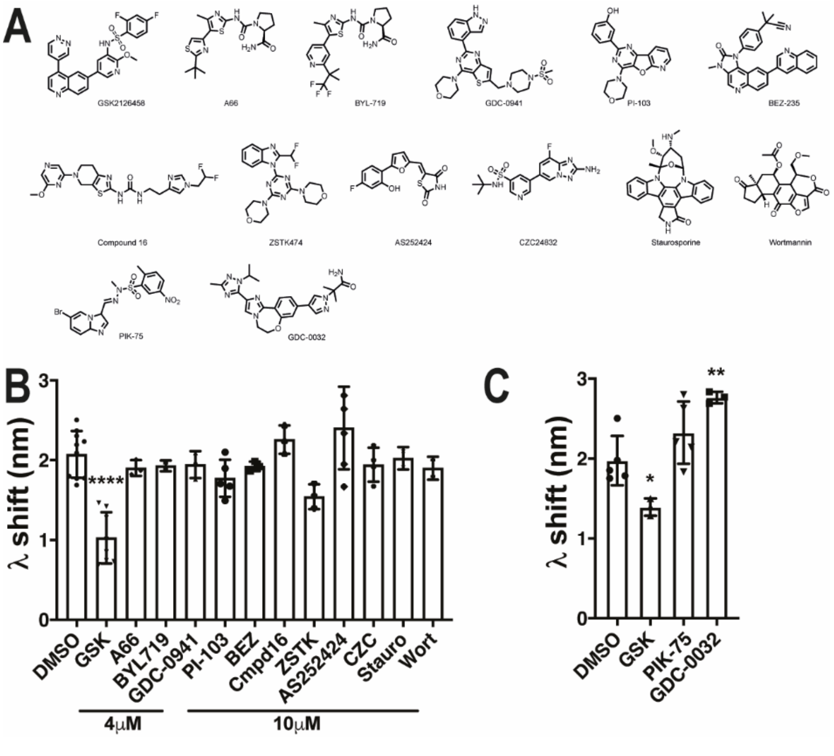

Table S4. Primer sequences for PI3Kα oncogenic mutations.

G106V_Forward	5' phospho GTC AAC CGT GAA GAA AAG ATC CTC AAT CGA 3'
G106V-Reverse	5' phospho TAC TGG TTC AAT TAC TTT TAA AAA GGG TTG 3'
G118D_Forward	5' phospho GAT TTT GCT ATC GGC ATG CCA GTG TGT G 3'
G118D_Reverse	5' phospho AAT TTC TCG ATT GAG GAT CTT TTC TTC ACG 3'
N345K_Forward	5' phospho AAA GTA AAT ATT CGA GAC ATT GAT AAG ATC 3'
N345K_Reverse	5' phospho CAC GTA GGT TGC ACA AAG AAT TTT TAT TCT G 3'
C420R_Forward	5' phospho CGT CCA TTG GCA TGG GGA AAT ATA AAC TTG 3'
C420R_Reverse	5' phospho GTG TTC CTC TTT AGC ACC CTT TCG GCC TTT AAC 3'
M1043I_Forward	5' phospho ATT AAT GAT GCA CAC CAT GGT GGC TGG ACA AC 3'
M1043I_Rev	5' phospho TTG TTT CAT GAA ATA CTC CAA AGC CTC TTG 3'
G1049R_Forward	5' phospho CGT GGC TGG ACA ACA AAA ATG GAT TGG ATC 3'
G1049R_Reverse	5' phospho ATG GTG TGC ATC ATT CAT TTG TTT CAT GAA ATA CTC 3'

Table S5. Protein preparation protocols for each figure.

Figure	Purification protocol	Storage buffer	Enzyme
2B, 2C	A		PI3Kα^{WT} , PI3Kα^{H1047R}
2D, 2E	B	Tris	PI3Kα^{WT} , PI3Kα^{H1047R}
3A, 3B	B	Tris	PI3Kα^{WT} , PI3Kα^{H1047R}
4A, 4B	B	Tris	PI3Kα^{WT} , PI3Kα^{H1047R}
5A,	B	Hepes	PI3Kα^{WT}
5B, 5D	B	Tris	PI3Kα^{WT}
Fig 6	B	Tris	PI3Kα^{WT}, PI3Kα^{E545K}, PI3Kβ, PI3Kδ
Fig 7	B	Tris	PI3Kα^{WT}
S1A, S1B, S1C, S1D	B	Tris	PI3Kα^{WT} , PI3Kα^{H1047R}
S2A, S2B, S2C, S2D, S2E, S2F, S2G, S2H	B	Tris	PI3Kα^{WT} , PI3Kα^{H1047R}
S6	B	Tris	PI3Kα^{WT} , PI3Kα^{H1047R}
S7	B	Tris	PI3Kα^{WT}
Fig S9			
Fig S10	B	Tris	PI3Kα^{G106V}, PI3Kα^{G118D}, PI3Kα^{N345K} , PI3Kα^{C420R} , PI3Kα^{E545K} , PI3Kα^{M1043I} , PI3Kα^{G1049R}
Fig S11	B	Tris	PI3Kα^{WT}
Table S3	B	Tris	PI3Kα^{WT}

Compound synthesis

Materials and Methods

Synthesis of 2,4-difluoro-*N*-(2-methoxy-5-(8-(pyridazin-4-yl)naphthalen-2-yl)pyridin-3-yl)benzenesulfonamide (**6**).

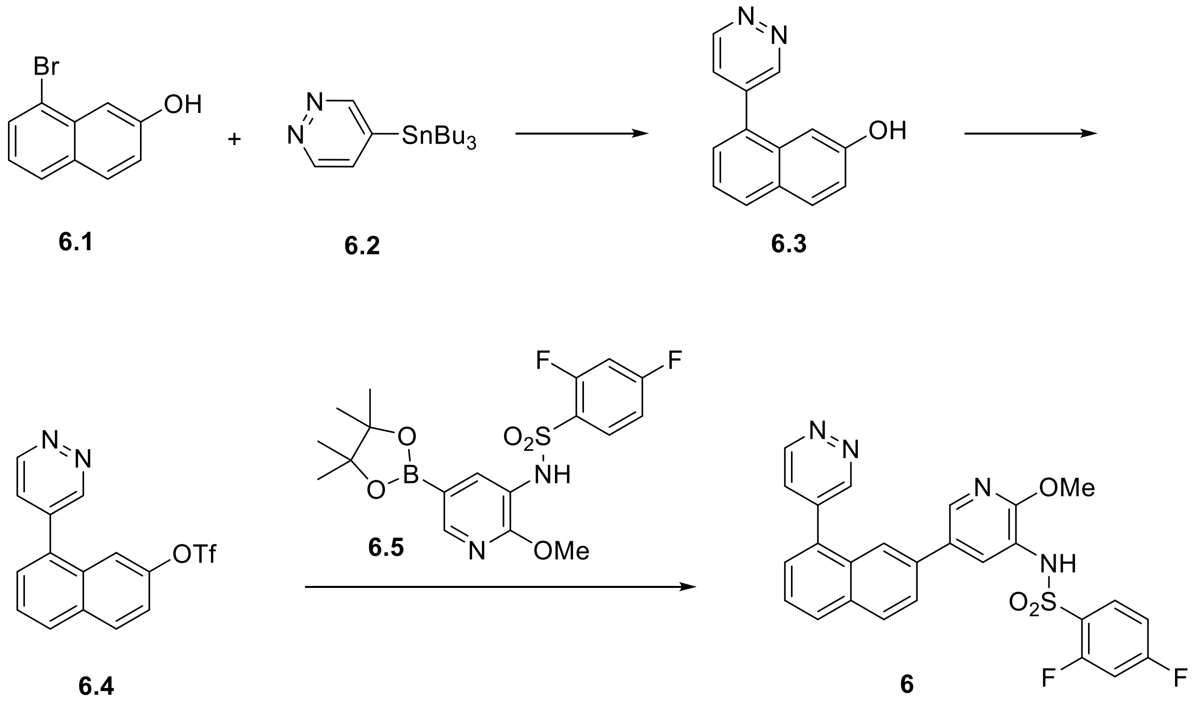

N_2 gas was bubbled through a solution of 8-bromonaphthalen-2-ol (**6.1**) (200 mg, 0.90 mmol) and 4-(tributylstannyl)pyridazine (**6.2**) (331 mg, 0.90 mmol) in dry dioxane (10 mL) for ca. 2 mins. Then $PdCl_2$(dppf) (146 mg, 0.18 mmol) was added and the resulting mixture was refluxed for 18 h under a balloon of N_2. The solvent was removed under vacuum, and the residue was chromatographed on silica (eluting with hexanes: EtOAc 2:1 to 1:1 to 1:2) to give 8-(pyridazin-4-yl)naphthalen-2-ol (**6.3**) as a pale brown solid (176 mg, 88%): ^{1}H NMR (d_6-DMSO) δ 9.87 (s, 1H), 9.39 (dd, *J* = 8.86, 1.23 Hz, 1H), 9.39 (t, *J* = 1.21, 1.21 Hz, 1H), 7.95 (d, *J* = 8.0 Hz, 1H), 7.91 (d, *J* = 8.9 Hz, 1H), 7.86 (dd, *J* = 5.2, 2.4 Hz, 1H), 7.47 (dd, *J* = 7.1, 1.3 Hz, 1H,), 7.41 (dd, *J* = 8.0, 7.1 Hz, 1H), 7.15 (dd, *J* = 8.8, 2.4 Hz, 1H), 7.02 (d, *J* = 2.3 Hz, 1H); MS (APCI$^+$) 223.1 (MH$^+$).

A solution of **6.3** (160 mg, 0.72 mmol) and pyridine (0.12 mL, 1.48 mmol) in dry CH_2Cl_2 (10 mL) was cooled to 0 °C. Triflic anhydride (0.15 mL, 0.89 mmol) was added over ca. 5 mins, and the reaction mixture was stirred for a further 1 h at 0 °C and then 2 h at

room temperature. The reaction mixture was diluted with water and extracted twice with CH_2Cl_2. The combined organic layers were dried (Na_2SO_4) and the solvent removed in vacuo. Chromatography on silica (eluting with hexanes: EtOAc 3:1 to 2:1 to 1:1) gave 8-(pyridazin-4-yl)naphthalen-2-yl trifluoromethanesulfonate (**6.4**) as a brown oil (26 mg, 10%). ^{1}H NMR ($CDCl_3$) δ 9.36-9.41 (m, 2H), 8.03-8.10 (m, 2H), 7.71 (dd, J = 8.2, 7.2 Hz, 1H), 7.66 (d, J = 2.3 Hz, 1H), 7.63 (dd, J = 5.1, 2.4 Hz, 1H), 7.58 (dd, J = 7.2, 0.8 Hz, 1H), 7.49 (dd, J = 9.0, 2.4 Hz, 1H); MS (APCI) 355.1 (MH^+).

N_2 gas was bubbled through a suspension of **6.4** (26 mg, 0.073 mmol) and 2,4-difluoro-*N*-(2-methoxy-5-(4,4,5,5-tetramethyl-1,3,2-dioxaborolan-2-yl)pyridin-3-yl)benzenesulfonamide (**6.5**) (S4) (47 mg, 0.11 mmol) in aqueous K_2CO_3 (2 mol L^{-1}, 1 mL) and DMF (2 mL) for ca. 2 mins. Then $PdCl_2$(dppf) (6.0 mg, 7.3 μmol) was added and the resulting mixture heated to 90 °C for 2 h under a balloon of N_2. The solvents were removed in vacuo. Chromatography on neutral alumina (eluting with CH_2Cl_2: MeOH 99:1 to 98:2 to 97:3) followed by crystallisation from MeOH-H_2O gave 2,4-difluoro-*N*-(2-methoxy-5-(8-(pyridazin-4-yl)naphthalen-2-yl)pyridin-3-yl)benzenesulfonamide (**6**) as an off-white solid (18 mg, 49%): ^{1}H NMR (d_6-DMSO) δ 10.30 (1H, s), 9.50 (dd, J = 2.4, 1.2 Hz, 1H), 9.40 (dd, J = 5.3, 1.2 Hz, 1H), 8.36 (1H, s), 8.19 (d, J = 8.4 Hz, 1H), 8.15 (d, J = 7.9 Hz, 1H,), 7.99 (dd, J = 5.3, 2.4 Hz, 1H), 7.91-7.85 (m, 3H), 7.75-7.63 (m, 3H), 7.55 (td, J = 8.8, 2.0 Hz, 1H), 7.17 (td, J = 12.6, 2.0 Hz, 1H), 3.65 (s, 3H, s); ^{13}C NMR (d_6-DMSO) δ 160.7, 160.5, 158.1, 158.0, 157.4, 152.1, 151.5, 142.6, 138.0, 135.1, 133.7, 133.4, 132.6, 131.8, 131.7, 130.2, 129.7, 129.5, 129.4, 128.7, 127.3, 126.0, 125.4, 121.5, 111.9, 111.7, 106.0, 105.8, 105.5, 53.4; HRMS Calcd. for $C_{26}H_{19}F_2N_4O_3S$: ($M+H^+$) m/z 505.1140; Found: m/z MH^+ 505.1142.

Synthesis of 2,4-Difluoro-*N*-(2-methoxy-5-(5-(6-methyl-7-oxo-6,7-dihydro-5*H*-pyrrolo[3,4-*b*]pyridin-3-yl)thiophen-2-yl)pyridin-3-yl)benzenesulfonamide (7). Compound **7** was prepared according to procedures described by Spicer et al (S5) and was isolated as a yellow solid, mp (MeOH/CH_2Cl_2) 261-264 °C (dec.): ^{1}H NMR (d_6-DMSO) δ 9.03 (1 H , d, J = 2.1 Hz), 8.28 (1 H, d, J = 2.1 Hz), 7.83-7.90 (1 H, m), 7.73 (1 H , d, J = 3.8 Hz), 7.70 (1 H, d, J = 2.3 Hz), 7.44 (1 H, d, J = 2.3 Hz), 7.24 (1 H, d, J = 3.8 Hz), 7.19 (1 H , ddd, J = 9.7, 9.7, 2.5 Hz), 7.09 (1 H , ddd, J = 8.4, 8.4, 2.2 Hz), 4.52 (2 H , s),

3.77 (3 H, s), 3.12 (3 H , s). Anal. Calcd. for $C_{24}H_{17}F_2N_4NaO_4S_2.H_2O$: C; 50.7, H; 3.4; N, 9.39. Found: C; 50.7, H; 3.2; N, 9.8.

References

S1 Gong, G. Q., Kendall, J. D., Dickson, J. M. J., Rewcastle, G. W., Buchanan, C. M., Denny, W. A., et al. (2017) Combining properties of different classes of PI3Kalpha inhibitors to understand the molecular features that confer selectivity. Biochem J. **474**, 2261-2276 10.1042/BCJ20161098

S2 Dornan, G. L., Stariha, J. T. B., Rathinaswamy, M. K., Powell, C. J., Boulanger, M. J. and Burke, J. E. (2020) Defining How Oncogenic and Developmental Mutations of PIK3R1 Alter the Regulation of Class IA Phosphoinositide 3-Kinases. Structure. **28**, 145-156 e145 10.1016/j.str.2019.11.013

S3 Collier, P. N., Messersmith, D., Le Tiran, A., Bandarage, U. K., Boucher, C., Come, J., et al. (2015) Discovery of Highly Isoform Selective Thiazolopiperidine Inhibitors of Phosphoinositide 3-Kinase gamma. J Med Chem. **58**, 5684-5688 10.1021/acs.jmedchem.5b00498

S4 Li, L., Zhang, H., Zhang, M., Zhao, M., Feng, L., Luo, X., et al. (2017) Discovery and Molecular Basis of a Diverse Set of Polycomb Repressive Complex 2 Inhibitors Recognition by EED. PLoS One. **12**, e0169855 10.1371/journal.pone.0169855

S5 Spicer, J. A., Miller, C. K., O'Connor, P. D., Jose, J., Huttunen, K. M., Jaiswal, J. K., et al. (2017) Benzenesulphonamide inhibitors of the cytolytic protein perforin. Bioorg Med Chem Lett. **27**, 1050-1054 10.1016/j.bmcl.2016.12.057
